# Urethral Catheter Biofilms Reveal Plasticity in Bacterial Composition and Metabolism and Withstand Host Immune Defenses in Hypoxic Environment

**DOI:** 10.3389/fmed.2021.667462

**Published:** 2021-06-23

**Authors:** Yanbao Yu, Harinder Singh, Tamara Tsitrin, Shiferaw Bekele, Yi-Han Lin, Patricia Sikorski, Kelvin J. Moncera, Manolito G. Torralba, Lisa Morrow, Randall Wolcott, Karen E. Nelson, Rembert Pieper

**Affiliations:** ^1^J. Craig Venter Institute, Rockville, MD, United States; ^2^J. Craig Venter Institute, La Jolla, CA, United States; ^3^Southwest Regional Wound Care Center, Lubbock, TX, United States

**Keywords:** proteomics, catheter biofilm, infection, bacterial metabolism, pathogen, polymicrobial, immune defense

## Abstract

Biofilms composed of multiple microorganisms colonize the surfaces of indwelling urethral catheters that are used serially by neurogenic bladder patients and cause chronic infections. Well-adapted pathogens in this niche are *Escherichia coli, Proteus*, and *Enterococcus* spp., species that cycle through adhesion and multilayered cell growth, trigger host immune responses, are starved off nutrients, and then disperse. Viable microbial foci retained in the urinary tract recolonize catheter surfaces. The molecular adaptations of bacteria in catheter biofilms (CBs) are not well-understood, promising new insights into this pathology based on host and microbial meta-omics analyses from clinical specimens. We examined catheters from nine neurogenic bladder patients longitudinally over up to 6 months. Taxonomic analyses from 16S rRNA gene sequencing and liquid chromatography–tandem mass spectrometry (LC-MS/MS)–based proteomics revealed that 95% of all catheter and corresponding urinary pellet (UP) samples contained bacteria. CB biomasses were dominated by *Enterobacteriaceae* spp. and often accompanied by lactic acid and anaerobic bacteria. Systemic antibiotic drug treatments of patients resulted in either transient or lasting microbial community perturbations. Neutrophil effector proteins were abundant not only in UP but also CB samples, indicating their penetration of biofilm surfaces. In the context of one patient who advanced to a kidney infection, *Proteus mirabilis* proteomic data suggested a combination of factors associated with this disease complication: CB biomasses were high; the bacteria produced urease alkalinizing the pH and triggering urinary salt deposition on luminal catheter surfaces; *P. mirabilis* utilized energy-producing respiratory systems more than in CBs from other patients. The NADH:quinone oxidoreductase II (Nqr), a Na^+^ translocating enzyme not operating as a proton pump, and the nitrate reductase A (Nar) equipped the pathogen with electron transport chains promoting growth under hypoxic conditions. Both *P. mirabilis* and *E. coli* featured repertoires of transition metal ion acquisition systems in response to human host-mediated iron and zinc sequestration. We discovered a new drug target, the Nqr respiratory system, whose deactivation may compromise *P. mirabilis* growth in a basic pH milieu. Animal models would not allow such molecular-level insights into polymicrobial biofilm metabolism and interactions because the complexity cannot be replicated.

## Introduction

Urethral catheter-associated urinary tract infection (CAUTI) is the most common type of complicated UTI. Enhanced risks of recurrence and spread to other organs (e.g., pyelonephritis and urosepsis) compared to acute urinary tract infections (UTIs) exist in nosocomial environments (1–3). Asymptomatic cases are diagnosed as catheter-associated asymptomatic bacteriuria (CAASB). The use of nearly 100 million urethral catheters per year worldwide, a 3–10% incidence of bacteriuria over 24 h following patient catheterization, and an average bladder catheter insertion time of 72 h (2) suggest that 9 million to 27 million CAUTI and CAASB cases occur globally per year. Indwelling Foley catheters are used by patients with anatomical and neurological urinary tract abnormalities and typically retained for 1–3 weeks before replacement to minimize microbial colonization.

Among the most common etiological agents of CAUTIs are *Escherichia coli, Klebsiella pneumoniae, Pseudomonas aeruginosa, Proteus mirabilis, Enterococcus*, and *Candida* spp. (1, 3, 4). These microbes have adapted to form biofilms on catheter surfaces; e.g., *Proteus* and *Providencia* spp. metabolize urea to NH_3_ and use it as a nitrogen source (3, 5, 6). Furthermore, urea degradation alkalinizes the pH of urine and triggers deposition of phosphate salt crystals in and on catheters, thus increasing the risks of luminal occlusion and complications such as urinary stones and kidney infection (3). Unless specific risk factors such as a compromised immune system exist, clinical guidelines do not recommend use of antibiotics in CAASB cases (7). Of major concern are acquired and innate resistances of bacteria causing CAUTI, many of these belonging to the multidrug-resistant group of gram-negative pathogens known as ESKAPE pathogens (8). Understanding mechanisms that underlie cooperation and competition of bacteria and fungi in urethral catheter biofilms (CBs) may lead to new approaches to prevent or target their formation.

Innate immunity has a key role in defending against bacteria invading the urinary tract. They express pathogen-associated molecular patterns recognized by immunoglobulin A and lipopolysaccharide-binding protein and then presented to Toll-like receptors on urothelial cells. Intracellular signaling pathways result in cytokine release followed by leukocyte infiltration. Evidence from murine studies verified that neutrophil recruitment is critical for bacterial clearance in the urinary tract (9). The mechanisms neutrophils use to resolve an infection include the secretion of effector proteins and peptides, the generation of reactive oxygen species, phagocytosis, and the formation of extracellular traps (10, 11). Bladder catheterization was also shown to cause sterile inflammation using a murine model. CD^45+^ neutrophils were the main type of infiltrating immune cells. In this model, *E. coli* and *Enterococcus faecalis* caused urothelial barrier disruption and further immune cell infiltration with pyuria (12). However, a proteomic study on samples from catheterized trauma patients was consistent with neutrophil infiltration at a higher level in individuals diagnosed with CAASB or CAUTI as compared to non-bacteriuric patients (13).

The pathogenesis of single-species–associated UTI and CAUTI has been extensively studied in the context of *E. coli* and *P. mirabilis* (4, 14–17). Type I fimbriae are thought to initiate mucosal colonization *via* their ability to bind to mannosylated uroplakins that coat the luminal surface of urothelial umbrella cells (18). *P. mirabilis* produces several types of fimbriae of which the best characterized ones are mannose-resistant Proteus-like (MR/P) fimbriae, uroepithelial cell adhesin (UCA), and proton motive force (PMF) fimbriae (16). Fimbriae are virulence factors involved in the formation of salt-encrusted (SE) CBs entailing struvite and apatite crystal deposition on catheter surfaces (3, 14, 16). Host proteins such as fibrinogen are also deposited on catheter surfaces (3, 4). Biofilm-embedded bacterial cells embedded disperse, swarm, and recolonize unoccupied catheter surfaces. Their ascendance to the kidneys poses an elevated threat of pyelonephritis and urosepsis (3). Microbial colonization dynamics in CBs over time are influenced by the availability of various nutrients such as nitrogen, carbohydrates, transition metal ions (TMIs), oxygen, and antibiotic drug exposure. Iron and zinc ions are specifically sequestered by the innate immune system during infection (14, 19, 20). CBs may have a mucoid phenotype derived from extracellular polysaccharides that encapsulate bacterial cells and impede phagocytosis. Furthermore, such biofilms have been associated with renal complications by blocking urine flow (3).

CBs typically harbor more than a single species (5, 6, 21). Culture-independent metagenomic analyses have highlighted the considerable microbial diversity in CBs, including fastidious bacteria, such as *Actinomyces, Stenotrophomonas, Finegoldia*, and *Corynebacterium* (22, 23). To what extent the entire microbial community elicits inflammatory responses by the host is unclear. A few studies have described biofilm profiles related to long-term catheterization. One study reported high prevalence of *E. coli, P. mirabilis*, and *Providencia stuartii* for bacteriuric patients catheterized over 4 or more weeks (24). In a survey of hundreds of urine samples from recurrently catheterized spinal cord–injured patients, the incidences of UTI with one and two or more bacterial species were 45 and 15%, respectively (25). Up to four co-colonizing pathogenic species were reported in the context of a study using 20 patients (26). Antibiotic drug treatments altered the composition of CBs and in many cases failed to clear the bacteria from the patients' urinary tracts (26).

Proteomic approaches enabling simultaneous surveys of pathogens and the host response to CAUTI, longitudinally in a set of patients, offer the unique opportunity to elucidate the dynamic interplay of cohabitant microbes in CBs and their responses to an activated immune system activation. In this report, we characterize microbial complexity and metabolism, as well as host–microbial dynamics on a protein level for bladder-catheterized spinal cord–injured patients.

## Results

### Human Subject Cohort and Specimen Collection Design

**Figure 1:**
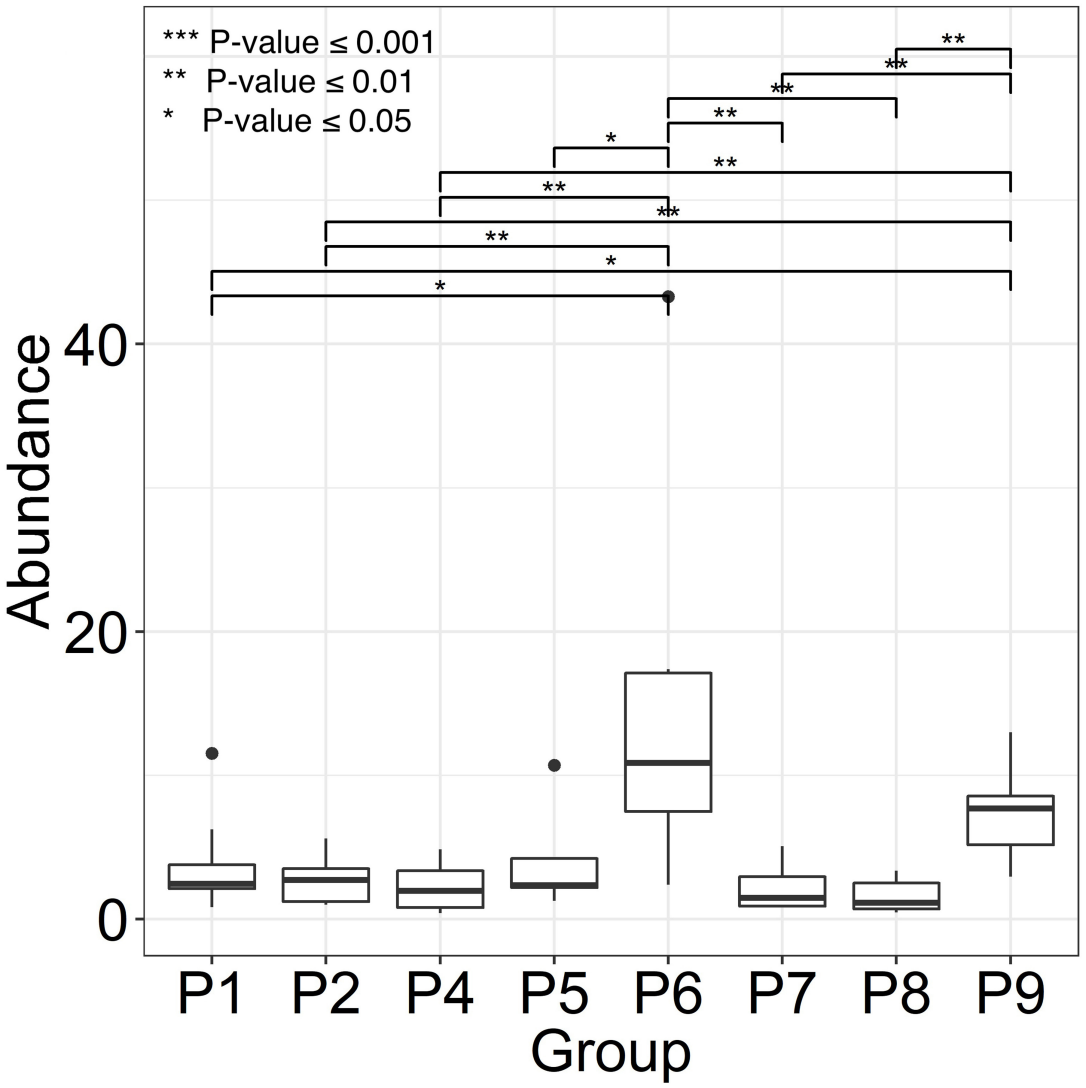
Variance in catheter biofilm (CB) biomasses derived from longitudinal specimen series of each patient. We use patient identifiers (P1 through P9) as in the main text, other Figures, and Supplementary Material. P3 was not included because of only two CB specimens. Biomasses were calculated as wet pellet weights in g × 10^−2^ for roughly 1.5-inch catheter pieces (*n* = 6–8). Salt deposits, if any, were dissolved by PBS wash steps. The horizontal bars depict statistically significant weight differences comparing datasets. Significance levels are coded ***≤0.001, **≤0.01, and *≤0.05 (*P*-values) using the Wilcoxon rank sum test.

Two female and seven male individuals of Hispanic or Caucasian ethnicity with spinal cord injuries (SCIs) were enrolled in the study. All patients suffered from chronic wound infections, as well as neurogenic bladder syndrome, which required catheterization. Indwelling bladder catheters were replaced during clinical visits every 1–3 weeks. Whereas, topical wound treatments with antibiotic drugs were common, systemic antibiotic treatments were administered intermittently in only three patients during the time of enrolment. The observations related to catheter type and encrustation, as well as patient medical histories (P1 through P9), are presented in Supplementary Table 2. UTI symptoms were not reported by the patients consistent with diagnosis of CAASB. However, patient P6 acquired a renal infection several weeks after collection of the last sample, likely due to ascension of bacteria from the bladder to the kidneys. Urine was collected in catheter bags for up to 24 h prior to a clinical appointment and Foley catheters removed in the clinic by nursing staff. The terms UP (urinary pellet) and CB (catheter biofilm) are used here for cell extracts from urine samples and catheter surfaces with or without luminal deposits, respectively. One or both sample types (121 in total) were collected for a clinical visit over 2–6 months. Salt crystal deposits on catheters were observed for P4, P5, P6, and P7. Catheter midsections were extracted to obtain the biomass for further analysis. As shown in the box plots of Figure 1, P6 revealed the highest average CB biomass. Catheters from P2, P4, P7, and P8 featured low biomass variations comparing timepoints. We did not analyze UP biomass data due to lack of control over urine collection intervals and patient fluid intakes.

### 16S rRNA Gene Taxonomic Data Support the Recurrence of Microbial Communities on the Patients' Sequentially Replaced Catheters

**Figure 2:**
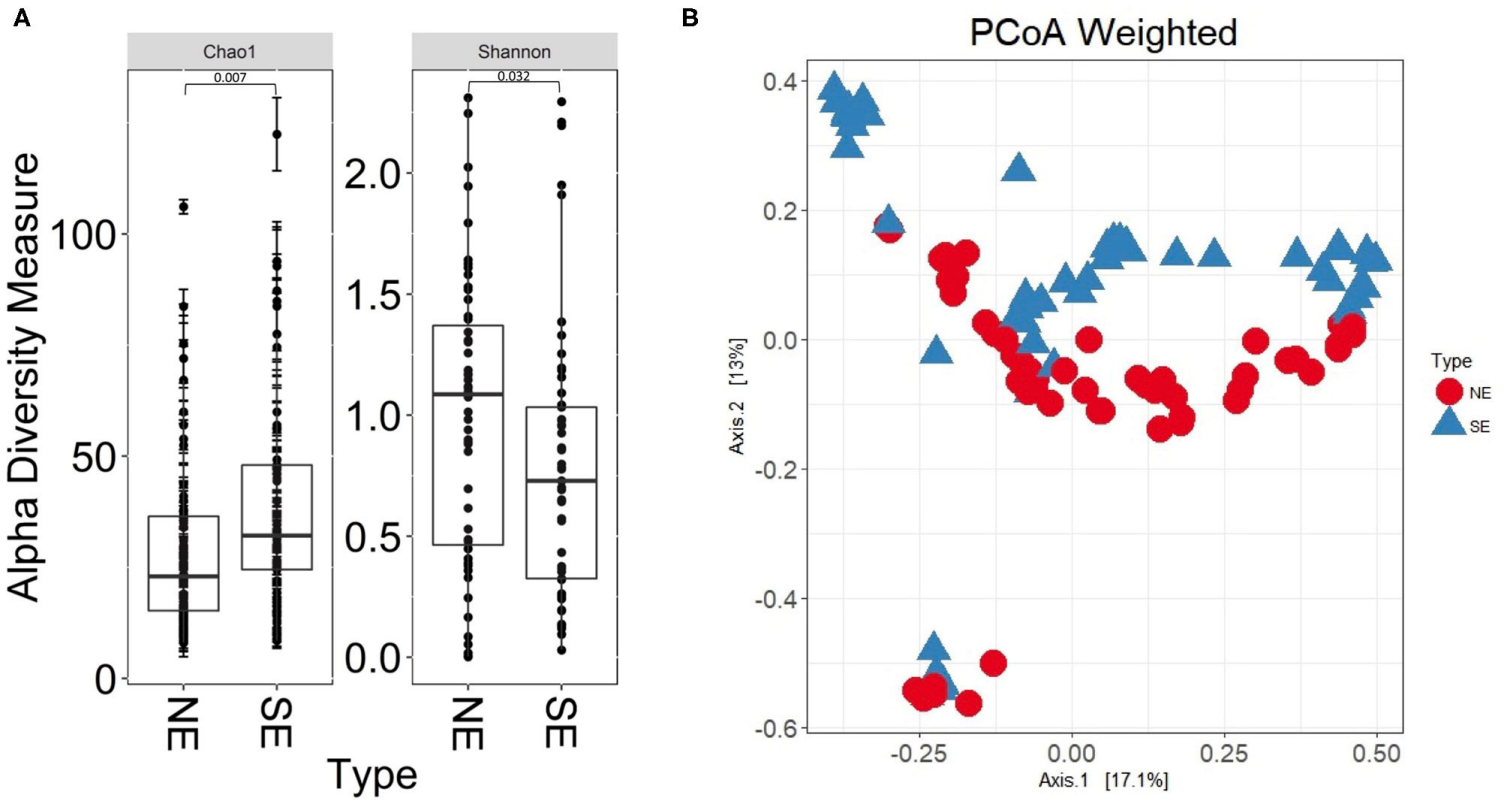
Bacterial α-diversity differences and principal component analysis comparing non-encrusted (NE) and salt-encrusted (SE) catheter 16S rRNA gene datasets. **(A)** α-Diversity difference calculations are based on Shannon and Chao indices, which account for abundance and evenness of operational taxonomic units. The 16S rDNA sequencing data from UP and CB samples associated with identical timepoints were separate entries. NE and SE diversities were statistically different based on Wilcoxon rank sum tests (*P* ≤ 0.05). **(B)** PCA separates the NE and SE groups.

The 16S rRNA gene profiles derived from sequencing of V1–V3 regions suggested bacterial colonization for all 112 UP and CB specimens; P9 samples were not available at the time of analysis. Supplementary Datasheet 1 lists all genus assignments and, for a subset of samples, data derived from microbial cultures. In-depth comparisons were available for two timepoints, 61UP and 54CB (P5 and P6, respectively). The samples were not frozen and preserved anaerobically in rich growth media. Extracts were cultured anaerobically and aerobically over 48 h, as reported (27). 16S rRNA taxonomic profiles and culture data showed good agreement for *Escherichia, Morganella, Aerococcus, Enterococcus*, and *Actinobaculum* (61UP) and *Citrobacter, Staphylococcus, Proteus*, and *Globicatella* (54CB). The fastidious anaerobes were of low to moderate abundances, including *Prevotella, Finegoldia*, and genera belonging to Actinobacteria and Fusobacteria (Supplementary Datasheet 1). Most bacteria appear to recolonize catheters following sequential replacement in the patient's urethra. The results are consistent with the urothelial retention of viable bacterial foci, even though bladder wash steps were administered in the clinic. As shown in Figure 2, the bacterial genus diversity was moderately lower for SE biofilms (P4, P6, and P7) compared to non-encrusted (NE) biofilms (P1, P2, P3, and P8). Principal component analysis (PCA) separated the NE and SE metagenomic profiles (Figure 2B). Competitive growth advantages of urease-producing *Proteus* and *Providencia* strains and bacterial coprecipitation with phosphate salts in alkalinized urine were reported (28). This may contribute to lower bacterial diversity in the CBs of SE profiles. The *Proteus* 16S rRNA gene indeed accounted for more sequence reads in P4, P6, and P7 (SE) samples than in P1 and P5 (NE) samples. Relative 16S rRNA gene abundance averages were 0.21 (σ = 0.28) and 0.012 (σ = 0.018), respectively.

### Proteomic Data Reveal Persistent Innate Immune Responses in the Patients' Urinary Tracts

**Figure 3:**
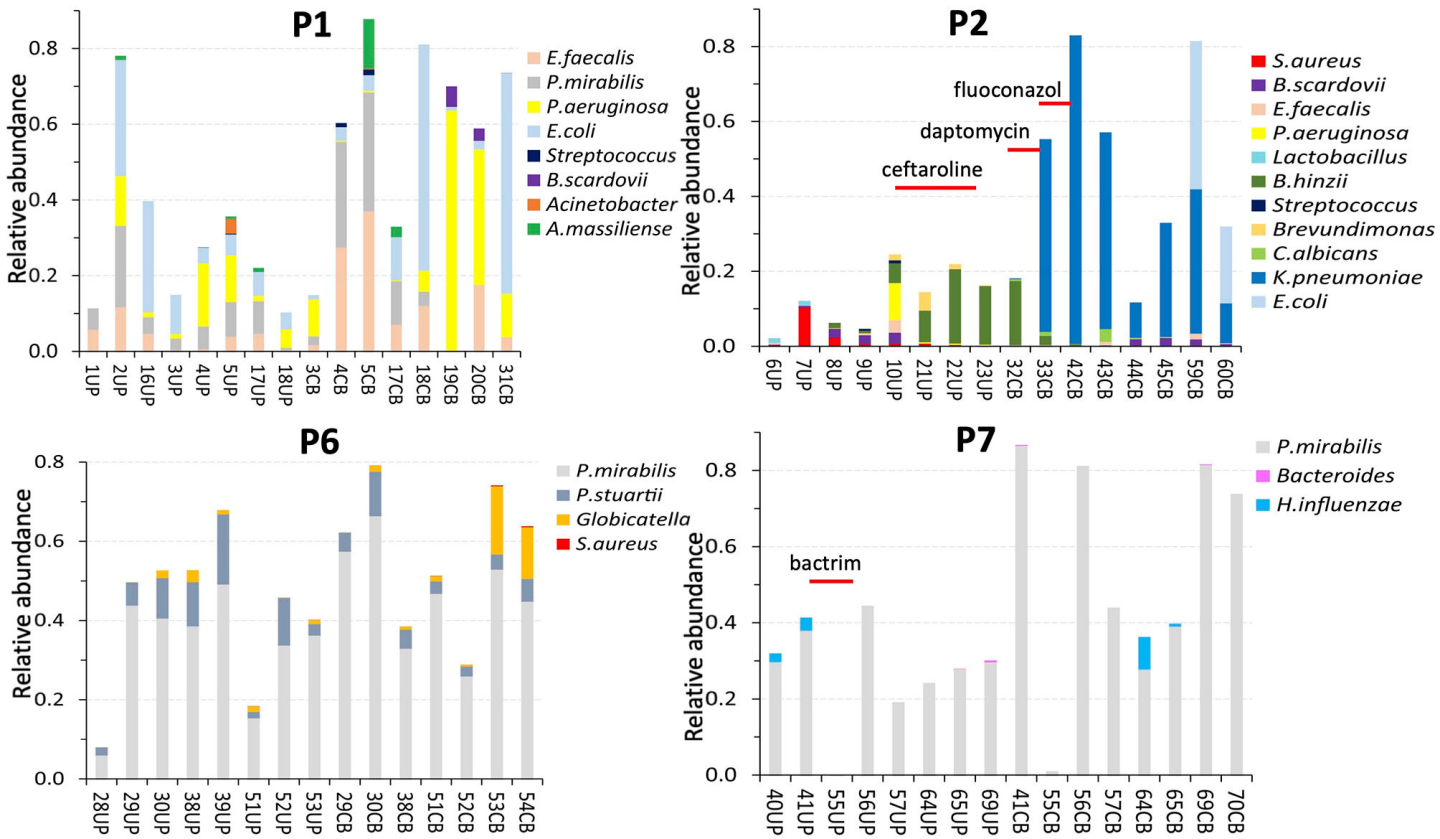
Profiles of microbial species according to metaproteomic analyses of samples from four patients. The number in the sample name (below the *x* axis) represents a specific collection timepoint. The name also includes the sample type (UP or CB) that we separated: UPs on the left and CBs on the right. The sample identifiers are unique regardless of patient. The patient identifier (P1, P2, P6, and P7) is inserted at the top of each diagram. The samples (and bars) are ordered from the first to the last collection timepoint, left to right. The time scale ranged from 7 to 23 weeks. Timepoint-matched CB and UP samples have the same number. The bar segment colors denote a species, or a genus in a few cases. The bar segment length represents relative abundance based on the sum of PSM counts for a species relative to PSM counts for the entire proteome. Thus, a bar with high values (e.g., 0.8) reflects a proteome made up of 80% PSMs of microbial origin and 20% PSMs of human origin. An inserted antibiotic drug name and a line underneath indicate a time when a patient was administered intravenously or orally with an antibiotic drug.

**Figure 4:**
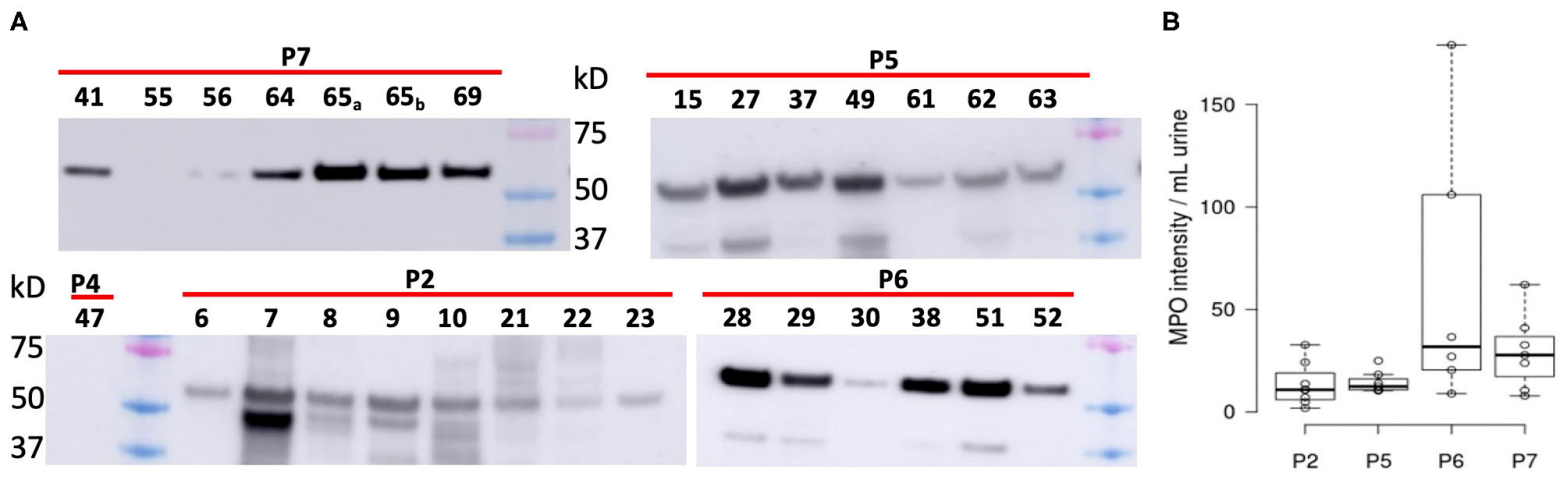
Differential analysis of myeloperoxidase from Western blot chemiluminescent band intensities (~55 kDa) of UP samples. **(A)** Western blot images pertaining to four patients. Loading was normalized for total protein amount per UP sample (~10–20 μg). We also display band intensities for samples where no pathogen was identified *via* proteomic analysis (P2, #6; P4, #47; P7, #55). Colored markers visualized in each image had *M*_r_ values of 75, 50, and 37 kDa (top to bottom). **(B)** Display of band intensities in box plots from the patients' UP samples (*n* = 6), with further normalization based on fractional volume applied from a lysate of a urine sediment.

We subjected 121 samples to proteomic analyses, and among them 42 collection timepoint-matched UP and CB samples. P3 data were not considered here because the number of samples was low preventing a meaningful longitudinal study. The numerical sample identifiers define specific collection timepoints and, with one exception in the P1 series, are chronological for a given patient. The time scale is depicted in the graphics of Figure 3 from left to right. Liquid chromatography–tandem mass spectrometry (LC-MS/MS) data simultaneously surveyed microbial and human proteomes. Heat maps (Supplementary Material) visualize the UP and CB human proteome profiles. Hierarchical clustering analysis revealing a prominent cluster of abundant proteins enriched in mature neutrophils supports the occurrence of pyuria in patients (7, 13). We identified human 8,000 proteins from samples derived from long-term catheterization of the urinary tract, the most extensive proteomic survey of clinical CB samples to date (Supplementary Datasheet 2). Analyzing the 62 UP datasets, five of the 15 most abundant proteins are neutrophil-specific and associated with the immune defense: myeloperoxidase (MPO), the iron-sequestering lactoferrin (LTF), and the calprotectin protein S100-A9, which sequesters zinc and contributes to inflammatory signaling. Western blot analyses of UP samples confirmed the presence of the heavy chain of MPO with an *M*_r_ of 55 kDa (Figure 4). Lower *M*_r_ bands (48 and 37 kDa) represent truncated versions, as also observed in a previous UTI study (10). The MPO abundance is normalized for total urinary protein concentration in the box plots of Figure 4. Samples from P6 and P7 had moderately higher MPO band intensities than those from P2 and P5. Correlation analyses for 15 proteins enriched in neutrophil granules (29) and two proteins enriched in eosinophils [based on Protein Atlas entries (30)] revealed good correlations (ρ > ~0.75) among neutrophil granule proteins, cathepsin G, azurocidin, elastase (NE), LTF, and MPO, and two eosinophil proteins, the bone marrow proteoglycan (PRG2) and eosinophil peroxidase. A correlation matrix is presented in the Supplementary Material. These data further support the notion that neutrophils and eosinophils infiltrate the urothelium followed by granule effector release to combat the pathogens in the CBs. In addition to keratins, we identified acute phase reactants among the 15 most abundant human proteins: complement factor C3, two fibrinogen subunits, and apolipoprotein B100 (Table 1). Acute phase responses are a consequence of immune cell–mediated inflammatory stimuli and involved in the resolution of infections.

**Table 1:** Proteins in UP samples that represent different cell types or innate immunity functions.

	**UniProt ID**	**Protein name**	**Quantity in UP proteome**	**Neutrophils (NG/NC), eosinophils (EG), UC/SEC, or APR^**∧**^**	**B/noB ratio^***$***^**	***P*-value (*t*-test)**
1	P07911	Uromodulin (UMOD)	5.282		0.940	0.25632
2^*^	P02788	Lactoferrin (LTF)	2.472	NG	3.295	0.00020
3	P13646	Keratin, type I cytoskeletal 13 (KRT13)	2.230	UC/SEC	0.812	0.48682
4	P01024	Complement C3 (C3)	1.882	APR	0.824	0.16925
5^*^	P05164	Myeloperoxidase (MPO)	1.863	NG	2.776	0.02514
6	P02675	Fibrinogen beta chain (FGB)	1.352	APR	0.664	0.23466
7	P01834	Immunoglobulin κ chain C region (IGKC)	1.347		0.680	0.16336
8	P02679	Fibrinogen gamma chain (FGG)	1.221	APR	0.711	0.22031
9	P19013	Keratin, type II cytoskeletal 4 (KRT4)	1.196	UC/SEC	1.010	0.29278
10	P02538	Keratin, type II cytoskeletal 6A (KRT6A)	1.035	UC/SEC	0.721	0.43854
11	P04114	Apolipoprotein B100 (APOB)	0.984	APR	1.083	0.30314
12	P01857	Immunoglobulin γ-1 chain C region (IGHG1)	0.919		1.669	0.46929
13	P08727	Keratin, type I cytoskeletal 19 (KRT19)	0.914	UC/SEC	0.721	0.31274
14^*^	P06702	Protein S100-A9 (S100A9)	0.912	NC	2.327	0.02243
15	P13647	Keratin, type II cytoskeletal 5 (KRT5)	0.831	UC/SEC	0.769	0.36410
16^*^	P20160	Azurocidin (AZU)	0.651	NG	10.264	0.00083
17^*^	P13727	Bone marrow proteoglycan (PRG2)	0.162	EG	4.632	0.00216
18^*^	P17213	Bactericidal permeability-increasing p. (BPI)	0.138	NG	2.855	0.00420
19^*^	P12724	Eosinophil cationic protein (RNASE3)	0.094	EG	6.940	0.00905
20^*^	P61626	Lysozyme C (LYZ)	0.129	NG	1.964	0.01857
21^*^	P08246	Neutrophil elastase (NE)	0.726	NG	3.948	0.01920

*The first 15 proteins were, on average, the most abundant proteins in UP sample datasets*.

* *Neutrophil and eosinophil effector proteins*.

∧ *Proteins localized in NG, neutrophil granules; NC, neutrophil cytoplasm; EG, eosinophil granules; UC/SEC, urothelial cells and squamous epithelial cells or part of the acute phase response (APR)*.

$ *Quantitative ratios of protein in UP samples with vs. without bacteriuria (B/noB)*.

*The t-test P-value (unequal variance, not corrected for multiple testing) pertains to the B/noB comparative protein abundance analysis*.

*T-test P-values pertain to differential protein abundances in the B/noB comparison*.

To determine if mechanical irritation from catheter insertion alone resulted in granulocyte infiltration, we differentially quantified human proteins from UP datasets with no or barely detectable bacteria (one sample each for P2, P4, and P7) and the other datasets. Two-tailed unequal variance *t-*tests revealed that 15 of 24 proteins with *P* < 0.05 were neutrophil or eosinophil effectors, which were less abundant in samples without bacteriuria (included in Supplementary Datasheet 2). The *t-*test results for nine effectors are listed in Table 1. Furthermore, Western blots showed faint or no MPO bands for the samples devoid of bacteria (UP6, UP47, and UP55; Figure 4). Hypoxia is known to occur in pathogen-infected tissues. In the proteomic datasets, we identified hypoxia up-regulated protein 1 and lactate dehydrogenase. Both proteins participate in cellular adaptations to low oxygen levels. Later, we refer to these hypoxic conditions to explain bacterial adaptations to the CB milieu.

### Pathogen Persistence Over Series of Catheter Replacements and Dominance of *P. mirabilis* Strains in SE CBs

We studied compositional and longitudinal changes over up to 16 timepoints by customizing metaproteomic searches for UP and CB sample series from eight patients to identify the full microbial repertoires. Profiles for four patients each are presented in Figure 3 and the Supplementary Material. The lengths of colored bar segments reflect quantitative contributions of a microbe to each sample. The LC-MS/MS–based data are generally unbiased, given that amplification and microbial cultures steps were absent. All microbial protein identities and normalized peptide-spectral match (PSM) counts are listed in Supplementary Datasheet 3. Only one fungal species, *Candida albicans*, was detected in samples from two patients (P2, P9). As fungi are difficult to lyse, *C. albicans* relative quantities in those cases may be underestimated. Longitudinal data series support the notion that catheters are colonized by bacteria that resided in the biofilms of previous catheters. Common uropathogens (*E. coli, P. mirabilis, K. pneumoniae, P. aeruginosa*, and *E. faecalis*) were prevalent and abundant in UP and CB samples. The observation of salt deposits on catheter surfaces, especially in the P4 and P6 samples, coincided with dominant urease-producing *P. mirabilis* strains. Catheter series that were not SE often depicted more dynamic microbial communities comparing timepoints, but timepoint series also revealed bacteria persistence (P1, P8, and P9). Systemic antibiotic drug treatments perturbed bacterial colonization with various posttreatment outcomes. The P2 profiles showed community replacement following treatment with two antibiotic drugs administered due to a wound infection (Figure 3). Ceftaroline led to the elimination of *P. aeruginosa, S. aureus, E. faecalis*, and *Bifidobacterium scardovii* from longitudinal UP samples (UP10–UP21). Daptomycin depleted *Bordetella hinzii*, while an extended-spectrum β-lactamase *K. pneumoniae* strain emerged as the dominant pathogen. A wound infection of P9 was treated with levofloxacin (75CB) and resulted in *Serratia marcescens* depletion from postantibiotic CBs, whereas *P. aeruginosa* and *E. faecalis* strains survived and persisted. These species were gradually replaced by *E. coli* and methicillin-resistant *Staphylococcus aureus* in the absence of further antibiotic drug intake (Supplementary Material).

We identified bacterial species rarely considered the main causes of UTI and CAUTI in several proteome datasets, among them fermentative Actinobacteria (*Actinobaculum massiliense, B. scardovii*, and *Propionimicrobium lymphophilum*) and non-fermenting opportunistic pathogens (*Brevundimonas diminuta, B. hinzii*, and *Stenotrophomonas maltophilia*). Their CB cohabitation is illustrated in Figure 3 and the Supplementary Material. The bacteria were detected over shorter time periods. Lactic acid bacteria (*E. faecalis, Aerococcus urinae*, and *Globicatella sanguinis*) were less abundant, although detected in many UP/CB samples. Comparing abundances of dispersed bacteria (UP samples) and biofilm-embedded bacteria (CB samples) for distinct collection timepoints, we examined occurrences of species-specific differences in dispersal of bacteria from CBs. *P*-values were lower than 0.05 for all species and all patients (Supplementary Material), suggesting that CB dispersal is not a process limited to only a subset of microbial species.

### Distinct *E. coli* and *P. mirabilis* Strains Are Dominant in Several Longitudinally Profiled CBs

**Figure 5:**
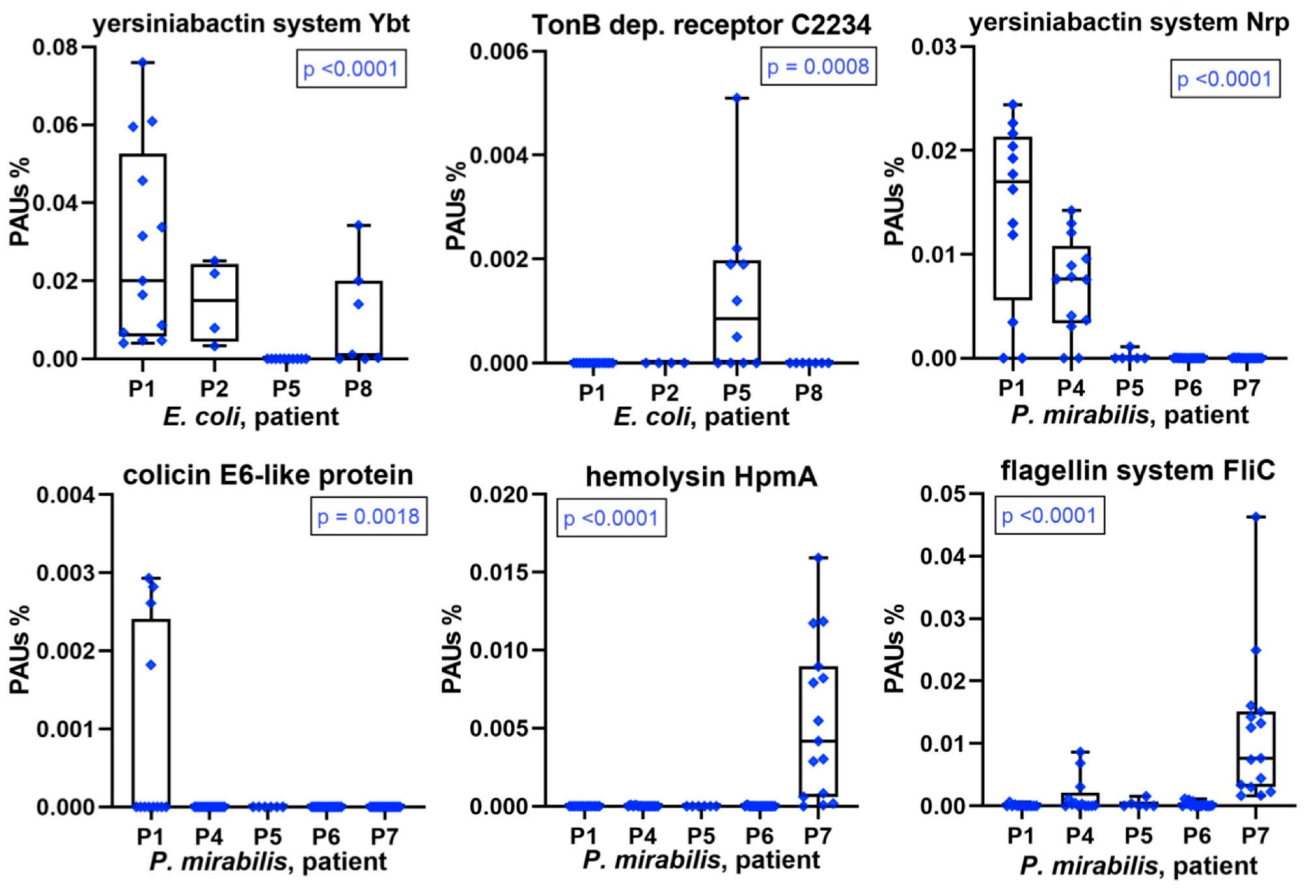
Patient-specific bacterial protein abundance differences derived from the PSM data for UP and CB samples (*P. mirabilis* or *E. coli*, as indicated). The proteins may represent a number of proteins that are part of a functional system (here, PSMs are the sum of all protein components). In the absence of a protein name, a gene ID is provided (e.g., C2234 for UTI89_C2234). The abundances are measured in proteomic abundance units (PAUs, see *Methods*). All proteins and systems reveal expression in only a subset of the patients. These data are indicative of the presence of a specific (or highly dominant) strain of the bacterial species. *P*-values provided here are based on non-parametric Kruskal–Wallis tests.

A repeatedly identified bacterial species in a longitudinal catheter series from a patient does not prove that specific strains recur. It could be a mixture of strains in each instance. Good proteomic coverage is required to support one or the other situation, and this was the case for *E. coli* and/or *P. mirabilis* in most patient datasets. We analyzed virulence factors, antibiotic resistance genes, and colicins, systems that are usually part of a pathogen's accessary genome limited to a subset of strains from a species. We found that some of these proteins or multiprotein systems were only identified in CB datasets of a subset of patients (Figure 5). Examples are the yersiniabactin system, which encodes the machinery for synthesis and export of a siderophore to capture host extracellular Fe^3+^/Mn^2+^ ions. Each system (Nrp in *P. mirabilis* and Ybt/Irp in *E. coli*) includes an outer membrane receptor for yersiniabactin uptake (PMI2596 and FyuA, respectively). A predicted *P. mirabilis* colicin E6 (PMIP30), aminoglycoside *O*-phosphotransferase APH (3′) conferring aminoglycoside antibiotic resistance (both encoded by a plasmid gene), and hemolysin HpmA were observed in proteome profiles of a single patient. *E. coli* cloacin CeaC and a siderophore/colicin receptor (C2234) were detected in CBs of only some patients (Figure 5 and Supplementary Datasheets 4D, 5E,G). Colicin E6 and cloacin are bacteriocins produced by some strains and bactericidal to other strains of the same species and serve to outcompete the latter in an ecological niche. Both were unique to CBs from patient P1. Colicin E6 and CeaC were also coexpressed with colicin immunity proteins in P1 datasets. We cannot rule out that low abundance strains of *E. coli* and *P. mirabilis* co-colonized the CBs with a dominance of the respective species in a given patient. Bacteriocins and high-affinity TMI/siderophore import systems may give the dominant strains a competitive advantage and ensure maintaining this dominance over a series of CB replacements in the infected patient.

The aforementioned single-strain dominance in a patient-specific manner is corroborated by hierarchical clustering data of *E. coli* or *P. mirabilis* proteomes included in the Supplementary Material. Especially, profiles with comprehensive proteome coverages such as *P. mirabilis* proteomes for P4, P6, and P7 clustered together.

### Competition of Bacteria for Iron and Other TMIs in CBs

**Figure 6:**
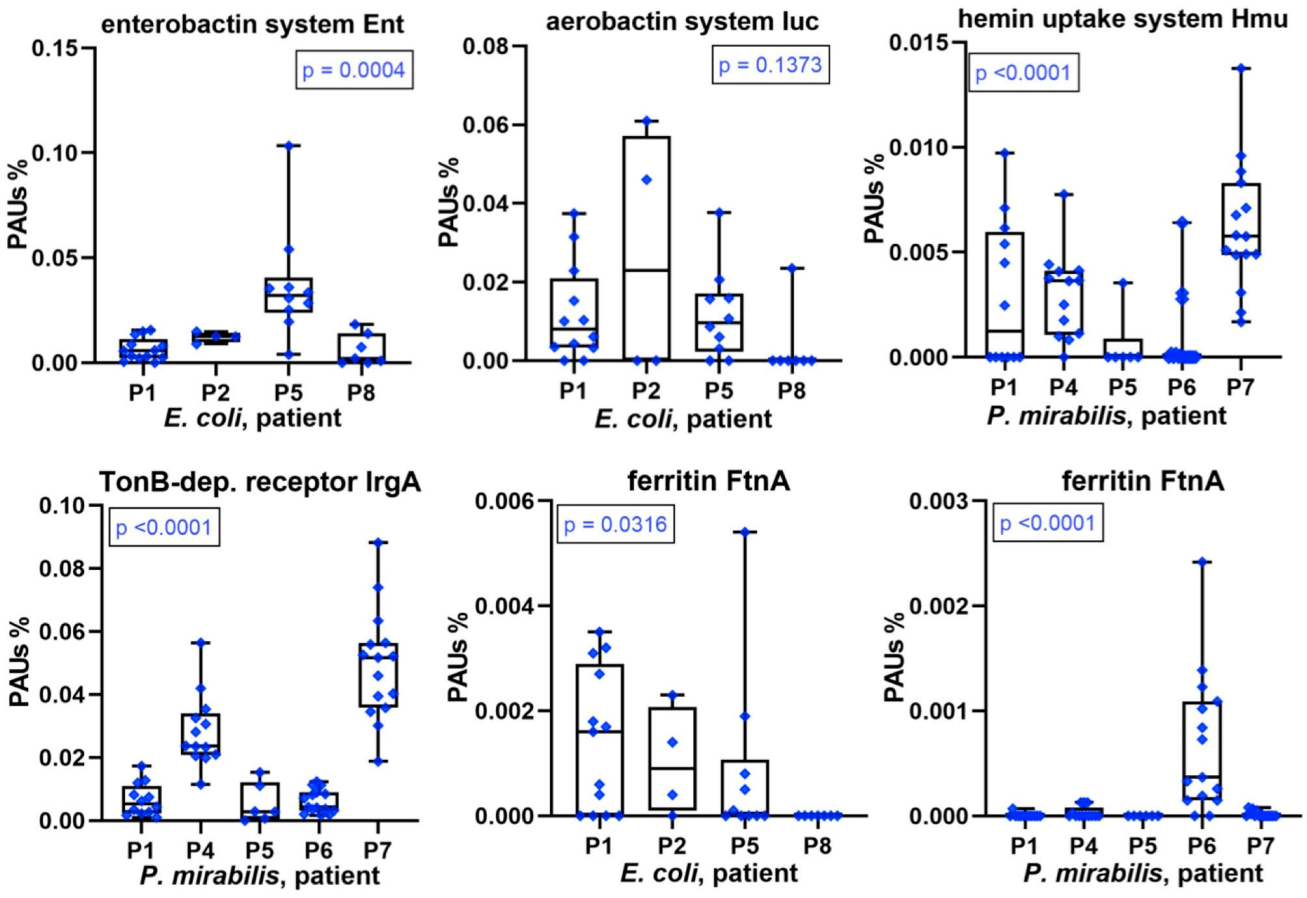
Patient-specific bacterial protein abundance differences derived from the PSM data for UP and CB samples (*P. mirabilis* or *E. coli*, as indicated). General descriptions of the Figure 5 legend apply. All proteins and systems included here pertain to transition metal ion acquisition of the bacteria or to iron storage (Ftn).

The scarcity of and competition for TMIs, especially iron, affect bacterial growth in the human host environment. Sequestering TMIs is a central part of nutritional immunity, and we described the high abundances of proteins involved in this process (LTF, calprotectin) for the human proteomes surveyed in UP samples. Here, we examined the diversity and relative abundances of bacterial TMI acquisition systems with high proteome coverage across series of CBs (*P. mirabilis* and *E. coli*). We mentioned yersiniabactin-like siderophore systems in the previous section. Marked differences in abundance comparing datasets associated with different patients were also observed for other acquisition systems and receptors involved in TMI transport and siderophore uptake. For *E. coli*, this included enterobactin and aerobactin synthesis and transport systems (Figure 6), the Chu heme uptake system, and the Sit Fe^2+^ ABC transporter. For *P. mirabilis*, this included TonB-dependent receptor potentially involved in Fe^3+^/siderophore uptake, the Hmu heme uptake system (Figure 6), and known and predicted metal ABC transporters (Sit, Feo, and PMI2957-PMI2960). An orphan receptor (IrgA/PMI0842, Figure 6), reported to be a virulence factor in some *P. mirabilis* UTI models (31), was a highly abundant protein in P7 and P4 datasets compared to those of other patients. Differential abundance profiles for additional proteins with roles in TMI import are displayed in Supplementary Datasheets 4D, 5E. The data underscore the multipronged strategy of both pathogens to acquire metal ions sequestered by the human host. Lower expression levels of TMI uptake and transport systems pertain to proteomes in P8 (*E. coli*) and P6 (*P. mirabilis*). The question arose as to whether that correlated with indicators of higher intracellular iron concentration, such as iron storage protein abundances. In the *E. coli* context (P8 data), the combined Bfr and FtnA amounts were lower than in proteomes from other patients. In contrast, Bfr and FtnA amounts in the *P. mirabilis* context were strongly elevated compared to other patients (Figure 6), suggesting that high bacterial iron supply may have allowed *P. mirabilis* to grow faster in P6 biofilms (see biomasses in Figure 1). That inference was supported by higher abundances of positively Fur-regulated proteins (SodA and AcnA) in *Proteus* cells of P6 compared to P1, P4, P5, and P7 (Supplementary Datasheet 5I). Iron abundance also affects energy metabolism pathways that are presented in the following paragraph.

### Metabolic Consequences of Low Iron and Oxygen Supplies in *E. coli* and *P. mirabilis* Residing in CBs

**Figure 7:**
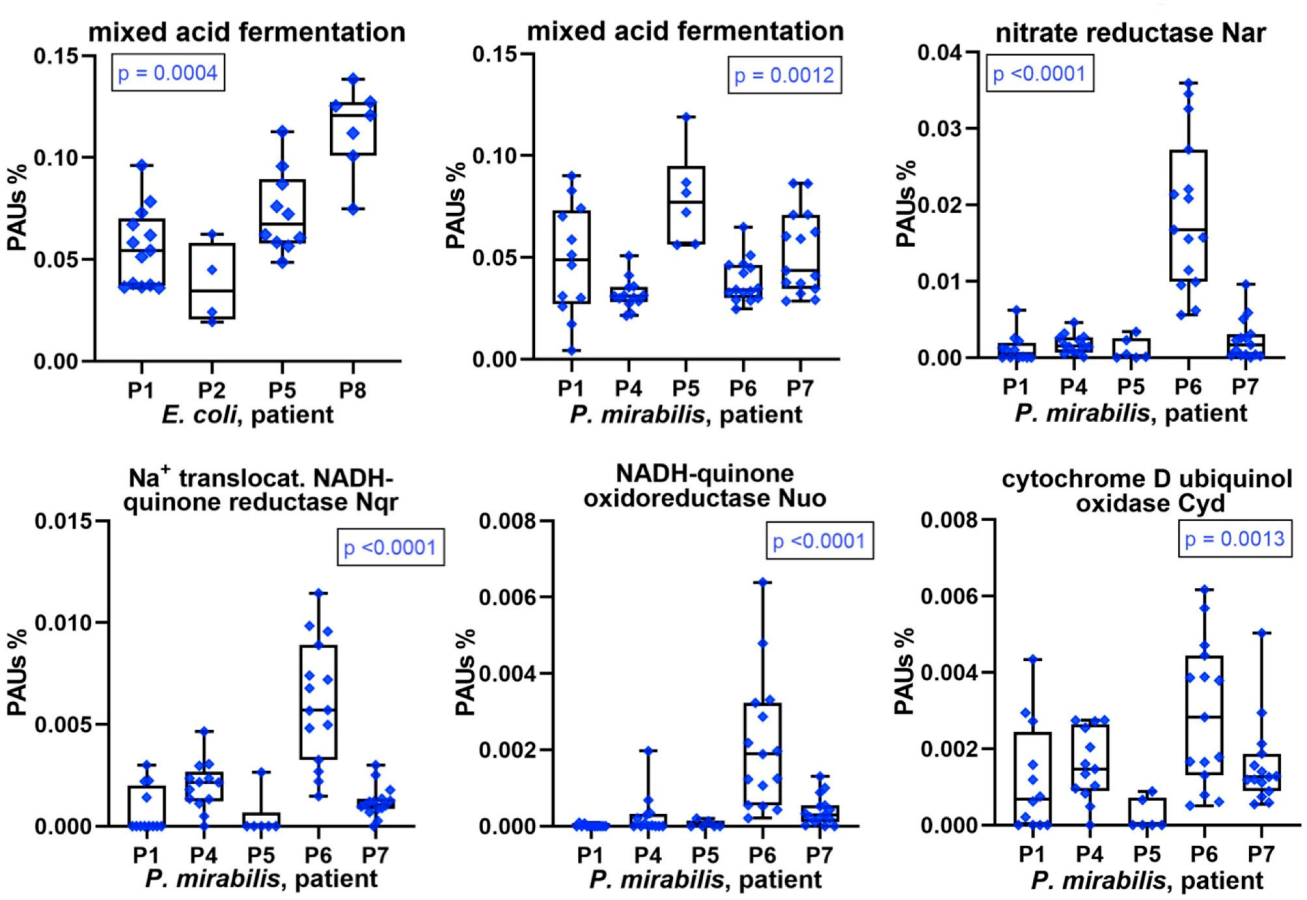
Patient-specific bacterial protein abundance differences derived from the PSM data for UP and CB samples (*P. mirabilis* or *E. coli*, as indicated). General descriptions of the Figure 5 legend apply. All proteins and systems included here pertain to energy metabolism including anaerobic and aerobic conditions.

We conducted differential quantitative analyses for enzymes, or a combination of enzymes, involved in energy metabolism in *E. coli* and *P. mirabilis*. Hypoxia in inflamed urothelial tissues and lack of oxygen penetration into deep layers of microbial biofilms suggest an important role of mixed acid fermentation (MAF) in the resident bacteria. The sum of abundances of enzymes involved in MAF was indeed high in most proteomes but varied comparing CBs from different patients (Figure 7). Enzymes included in the MAF group are provided in Supplementary Datasheets 4F, 5H. Relative MAF abundance levels were highest in *E. coli* biofilms from P8 followed by P5 and in *P. mirabilis* biofilms from P5 followed by P1 and P7. We note that *P. mirabilis* ferrous iron ABC transporter Sit, known to function as an iron transporter in an anaerobic milieu (32), was most highly expressed in proteomes of P1, P5, and P7. Generally, the need for iron cofactors in enzymes engaged in MAF is low compared to enzymes participating in the citric acid cycle and respiratory enzyme systems. In the two Enterobacteriaceae species, both anaerobic and microaerophilic respiratory systems were apparently active together with MAF. This included NADH:quinone oxidoreductases I (Nuo), formate dehydrogenases Fdo (*P. mirabilis*), and Fdn (*E. coli*) as electron donor systems and nitrate reduction I and VIII pathways [*via* the nitrate reductase I (Nar) and Nir enzymes] and cytochrome bd-I ubiquinol oxidase (Cyd) as electron acceptor systems, all of which are coupled to the proton-motive force. For *P. mirabilis*, there was strong evidence for differential abundances of these enzymes, all of which have Fe-S reaction centers comparing patient datasets (Figure 7 and Supplementary Datasheets 5H,I). In accordance with the predicted higher cytoplasmic iron supply in *P. mirabilis* cells of P6 biofilms, expression data suggest that the pathogen utilized the respiratory systems more extensively to facilitate rapid growth in the urothelial milieu. Interestingly, the NADH:quinone oxidoreductases II (Nqr) was more abundant than Nuo in *P. mirabilis* P6 and P4 biofilms. Nqr may be an efficient respiratory enzyme under hypoxic conditions in an alkalinized CB milieu, which is discussed later on. The sum of citric acid cycle enzyme abundances was highest in *P. mirabilis* proteome of P6 followed by P4. The enzymes provide reducing equivalents (NADH) for the membrane-associated Nuo and Nqr systems. To cope with oxidative stress derived from the use of Fe-S reaction centers and host enzymes such as MPO, several peroxidases and superoxide dismutases were highly expressed by *E. coli* and *P. mirabilis* (Supplementary Datasheets 4G, 5I). To summarize, *P. mirabilis* and *E. coli* succeed in regulating and mobilizing energy generation pathways in dependence of iron starvation and hypoxic conditions in a polymicrobial biofilm and thus adapt to a hostile host milieu.

### Other Factors Promoting Fitness and Virulence of *E. coli* and *P. mirabilis* in CBs

**Figure 8:**
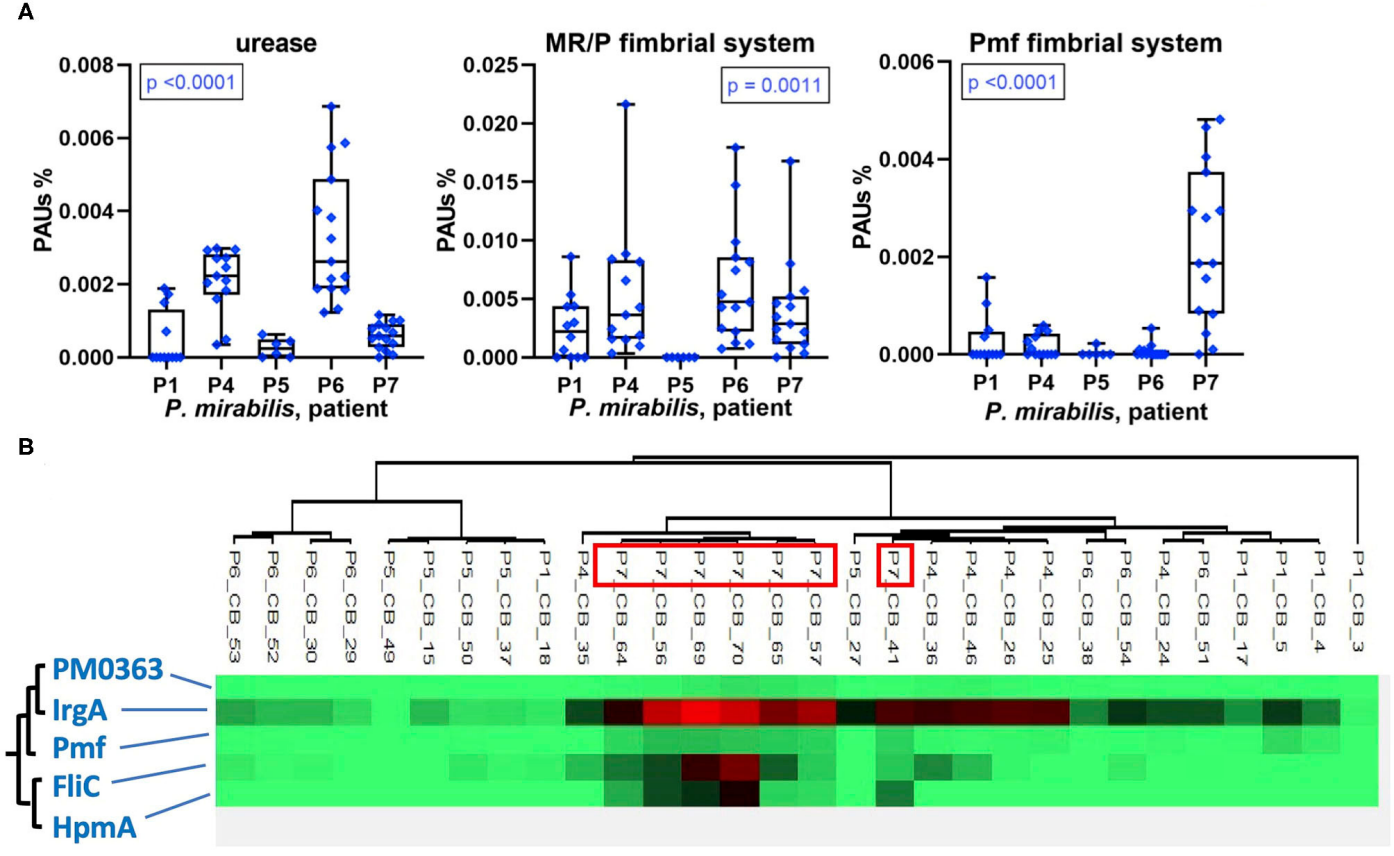
Patient-specific bacterial protein abundance differences derived from the PSM data for UP and CB samples (*P. mirabilis* or *E. coli*, as indicated) and evidence of a protein cluster abundant in patient P7. **(A)** General descriptions of the Figure 5 legend apply. Urease and MR/P fimbriae are well-characterized virulence factors. **(B)** Pmf, a less characterized *P. mirabilis* fimbrial system, reveals a pattern of coexpression with the hemolysin HpmA and the abundant TonB-dependent outer membrane receptor IrgA. The clustering analysis is based on Pearson correlations.

In addition to TMI acquisition systems, other virulence factors of uropathogenic *P. mirabilis* and *E. coli* strains have been characterized. We identified some of these in UP and CB datasets. The epithelial adhesion-associated factors HEK (only in P1) and type 1 fimbriae Fim (in P1, P2, and P8) were detected in *E. coli* proteomes in low and high quantities, respectively (Supplementary Datasheet 4B). The abundances of *P. mirabilis* urease varied comparing CB datasets from distinct patients and revealed a pattern similar to MR/P fimbriae: high quantities in CBs of P6 followed by P4 and the lowest average quantities in P5 (Figure 8). The robust *P. mirabilis* growth in P6 biofilms apparently facilitated by higher levels of respiration coincided with high urease and MR/P fimbrial expressions. Neither urease nor type 3 fimbriae were detected for a *P. stuartii* strain that cohabitated P6 biofilms. We examined proteomic data for additional coexpression patterns of known or putative virulence factors. Hierarchical clustering of quantities of 50 virulence and fitness-associated systems for *P. mirabilis* revealed a cluster of the cytotoxin HpmA, the flagellar apparatus Fli, the Pmf fimbriae, and the TonB-dependent receptor IrgA (Figure 8). These proteins' high abundances in P7 displayed in Figures 5, 6, 8 may contribute to urothelial cell internalization (see *Discussion*). In P7 biofilms, *P. mirabilis* relied more on MAF than on respiratory systems to generate cellular energy.

## Discussion

Our systems-level study on biofilms growing on indwelling catheters from neurogenic bladder patients generated new insights into polymicrobial complexity, longitudinal dynamics of pathogens colonizing latex/silicone catheters over series of catheter replacements (7–24 weeks and up to eight catheter replacements), and disruptions of CBs caused by systemic antibiotic drug treatments. *Via* proteomics, we obtained insights into transport and metabolic pathways for prevalent pathogens, *E. coli* and *P. mirabilis*, related to their metabolism, their utilization of scarce nutrients in the patients' urinary tracts, and their ability to persist over many months in serially replaced CBs. We emphasize that this study is unique in that it investigated polymicrobial communities organized as biofilms that truly existed in a human body niche. These data allowed insights that cannot be gained with animal models of CAUTI. The latter cannot mimic the complexity of polymicrobial CBs, do not reflect the human immune response, do not consider the chronicity of CAUTI that is observed in the spinal cord–injured patient population, and cannot mimic the clinical catheter replacement practice. To provide an example, gradients of microaerophilic to anaerobic conditions on catheter surfaces that reveal luminal salt deposits are impossible to mimic *via* murine CAUTI models. A disadvantage of our approach is the challenge to confirm most hypotheses resulting from UP and CB meta-omics profiles, e.g., by following up on the inferred importance of virulence factors or metabolic fitness of the uropathogens, as well as the cooperation of diverse bacterial species within CBs.

Culture-independent approaches previously identified fastidious bacteria in CBs in the context of CAUTI and CAASB (22, 23, 33). We confirmed anaerobic bacteria as cohabitants of CBs for six of the nine patients based on both 16S rRNA and proteomic profiles, including *A. massiliense, P. lymphophilum, B. scardovii, Veillonella parvula*, and *Campylobacter curvus*. Fastidious microbes tolerating oxygen (*A. urinae* and *G. sanguinis*) were identified in CBs, but less common compared to the genetically related *E. faecalis*. We characterized the metabolic states and mechanisms for nutrient acquisition within CBs for *A. massiliense, A. urinae*, and *G. sanguinis* using some of the specimens surveyed here (27, 34). These bacteria produce predicted TMI acquisition proteins but do not express systems to synthesize siderophores for high-affinity Fe^3+^ import. With an energy metabolism relying on fermentation, Fe-S cofactor needs are diminished as reported for *E. faecalis* (35). Our proteomic data in CBs for *E. faecalis* were not conclusive as it pertains to such iron import systems, as reported elsewhere *via* citrate/hydroxamate siderophores (35). An accepted paradigm is that infection sites are hypoxic due to high oxygen consumption of the inflamed epithelial and infiltrating immune cells and a vascular pathology diminishing local blood supply (36). Furthermore, biofilm layers at >50-μm depth lack oxygen penetration, as measured for *P. aeruginosa* (37), forcing facultatively anaerobic bacteria to alter their energy metabolism as shown for uropathogenic Escherichia coli (UPEC) (38). Our data are consistent with the notion that the aforementioned fermentative bacteria exploit the anaerobic niches within CBs.

Clearly most abundant and prevalent in the CBs were facultatively anaerobic bacteria with abilities to ferment and activate aerobic and anaerobic (nitrate) respiratory systems, such as UPEC, *P. mirabilis*, and other Enterobacteriaceae. However, bacteria thought to be obligate aerobes (*B. hinzii*) and non-fermenting bacteria that use denitrification to generate energy in a low-oxygen milieu (*P. aeruginosa* and *B. diminuta*) (39, 40) were also identified from several patients. It was striking that all but three UP/CB specimens showed evidence of colonization with opportunistic pathogens, after removing timepoints where patients knowingly underwent systemic antibiotic therapy. We also observed that the use of antibiotics resulted in different outcomes; in two cases (P2), it resulted in replacement of the CB community with bacteria not susceptible to the antibiotic; in two cases (P7 and P9), major colonizing strains regrew after antibiotic drug treatments ceased. It is plausible that genetically encoded resistance/tolerance elements and physical protection of bacteria in the biofilms contributed to these outcomes. Our data on the long-term persistence of uropathogens in CBs and on disruptions caused by antibiotic therapy were in good agreement with another study (26). High-resolution meta-omics analyses revealed that the persistence of bacteria profiled at high proteomic coverage was linked to distinct, dominant strains. These strains remained viable during constant challenge by immune cells including neutrophils, eosinophils, and their protein effectors and when catheters were substituted in the clinic by professional staff. Chronic innate immune responses were specifically linked to the actual presence of bacteria in CB and UP samples. In the few samples without evidence of pathogens, human proteomes were not enriched for granulocyte effectors. This contrasts with the finding of sterile inflammation upon catheterization of the urinary tract (12), but is in better agreement with a study that examined inflammatory protein profiles in short-term catheterized patients with and without evidence of asymptomatic bacteriuria or UTI (13).

Using proteomic data for two species well-represented in longitudinal CB sample series, we focused on the analysis of virulence factors and energy metabolism in *E. coli* and *P. mirabilis* strains in the hostile host environment. Fitness advantages based on the expression of urease by *P. stuartii* and, particularly, *P. mirabilis* have been described (24, 25). Our data confirm that *P. mirabilis* strains have significant advantages resulting in quantitative dominance in CBs at a high pH when catheters become encrusted with insoluble phosphate salts that establish salt-bacterial coaggregates and build large luminal deposits in an extracellular matrix (3, 16, 17, 26). We discovered that there is not a distinct *P. mirabilis* proteomic signature associated with high bacterial density and persistence in CBs. In contrast, strong protein expression differences in a patient-specific manner were observed. For instance, the cytotoxin HpmA was expressed only in P7, and hierarchical clustering analysis identified a pattern of similarly high abundances for three other systems: PMF fimbriae, the flagellar apparatus Fli, and the TonB-dependent receptor IrgA whose ligand is unknown. Although we did not conduct studies using cellular or *in vivo* models, invasion of urothelial cells has been previously linked to Fli-mediated mobility (31, 41). HpmA was attributed to pore-forming toxin activity. An orthologous cytolysin ShlA (*S. marcescens*) was reported to cause autophagy in epithelial cells predisposing them to bacterial internalization (42). IrgA (PMI0842) was reported to contribute to *P. mirabilis* fitness, but a definitive functional role was not identified (43). Pmf modulates the architecture of CBs *in vitro* (44). Especially double mutants of MR/P and Pmf fimbriae attenuated virulence in a UTI murine model (31). We speculate that these four proteins (or systems) cooperated in CBs to allow the dominant *P. mirabilis* strain in P7 to compromise and then invade urothelial cells, followed by reemergence, similar to the invasion–release cycle described for *E. coli* strains causing recurrent UTI (45, 46). The MR/P fimbriae, best characterized among a large repertoire of apparent fimbriae systems in *P. mirabilis* (44), revealed the highest quantities in P4 and P6, which also contained the highest quantities of urease. Given that the biomasses of CBs in P6 were especially large and that P6 advanced to a renal infection soon after specimen collection ended, our data corroborate the evidence that the two systems are important for pathogenicity of *P. mirabilis* in the human urinary tract, as previously reviewed (31).

TMI acquisition factors are important to pathogenicity, although their roles seem redundant so that deletion of a single system does not turn a mutant avirulent in most UTI models. We identified and quantified numerous TMI acquisition systems. Among those, yersiniabactin systems were expressed by *E. coli* and *P. mirabilis* strains (Ybt and Nrp, respectively) in a patient-specific manner. In other studies, yersiniabactin was found to act as proinflammatory factors inducing hypoxia-inducible factor 1α and confer survival advantages to *E. coli* within phagocytes by acting as a superoxide dismutase mimic based on copper reduction (47, 48). In our data, there was no evidence of higher inflammation or survival advantages in the urinary tract of patients where Ybt and/or Nrp systems were expressed. Remarkably, the *E. coli* strains produced three of four well-characterized systems for siderophore or hemin import in moderate to high abundance in P1, P2, and P5. Our data suggest that the substantial repertoire of systems for uptake and intracellular transport of TMIs, partially characterized in *E. coli* and *P. mirabilis* (49, 50), is relevant to fitness and survival in the human host.

Iron and oxygen flux in the hypoxic, iron-sequestering human host milieu are factors that strongly affect energy metabolism in these uropathogens. Quantitative proteomic data confirmed strong variations in the utilization of fermentation enzymes (MAF), anaerobic respiration using the pair of formate dehydrogenase (Fdo) and Nar, and microaerophilic respiration *via* the NADH:quinone oxidoreductases I (Nuo; in *E. coli*) and I and II (Nuo and Nqr; in *P. mirabilis*). These oxidoreductases are known to serve as electron donor systems, whereas the oxidase CydAB serves as an electron acceptor system in both species. *P. mirabilis* proteomes in P6 revealed high iron storage protein quantities (FtnA and Bfr), together with high abundances of respiratory enzymes, suggesting that iron availability in the cells was necessary (perhaps not sufficient) for the activity of respiratory systems to generate energy through the PMF. Respiratory systems depend on iron due to the functional necessity of Fe-S reaction centers. We hypothesize that luminal salt encrustation of catheters in P6 resulted in higher oxygenation allowing *P. mirabilis* to generate energy efficiently by employing anaerobic and microaerophilic respiratory pathways. Furthermore, we noticed a high quantity of Nqr, the NADH:quinone oxidoreductase that functions independent of a proton potential to allow respiration, in P6. The role of Nqr may be critical in the alkalinized milieu of encrusted urethral catheters facilitated by the degradation of urea to ammonia. Nuo may not work effectively in this alkaline milieu because electron transfer from NADH to oxygen is coupled to the generation of a proton-motive force with the translocation of four H^+^ ions per molecule NADH across the cytoplasmic membrane. Indeed, for *Vibrio cholerae*, a study has identified the key role of Nqr for the generation of transmembrane voltage and import of H^+^ by electrogenic H^+^/Na^+^ antiporters at a high pH and low osmolarity (51). It is intriguing to functionally characterize the potential advantage of utilizing the enzyme system Nqr for *P. mirabilis* energy metabolism in alkalinized urine and during encrustation of catheters. *E. coli* Nqr has been reported to be used preferentially for aerobic and nitrate respiration (52). We assume that the considerable iron starvation of the pathogens in CBs, given the induction of many Fur-repressed and repression of several Fur-activated proteins, may have triggered preferential energy generation *via* MAF, which is consistent with the previously reported iron-sparing response (53). We also infer that the high biomass of P6 biofilms and the renal infection of P6 are the consequence of more aggressive growth of *P. mirabilis* based on higher oxygenation in the encrusted catheter lumen. This is in agreement with the reported increased risk of renal CAUTI complications when biofilms form in the lumen of indwelling bladder catheters (54).

## Methods

### Ethics Statement

The Southwest Regional Wound Care Center (SRWCC) in Lubbock, TX, and the J. Craig Venter Institute (JCVI, Rockville, MD) created a human subject protocol and a study consent form (#56-RW-022), which were approved by the Western Institutional Review Board in Olympia, WA, followed by JCVI's IRB in 2013. All subjects were adults and provided written consent. The specimens were collected firsthand for the purpose of this study. There was a medical need to serially replace indwelling Foley catheters in patients because of their affliction with neurogenic bladder syndrome. Scientists at the JCVI did not have access to data allowing patient identification. Electronic and printed medical records were encrypted, retained at the clinical site for 4 years to facilitate integration of clinical and biological research data, and then destroyed.

### Human Subjects and Study Design

Nine subjects with irreversible SCIs were enrolled in this prospective study. They suffered from neurogenic bladder syndrome. Catheter replacement was part of routine patient care at SRWCC. Medical data included gender, ethnicity, antibiotic use, and the diagnosis of chronic wound infections and diabetes. Subjects provided 3–15 specimens (urethral catheters and urine from catheter collection bags) collected longitudinally in 1- to 4-week intervals, depending on the frequency of visits that the patient and physician agreed upon. Minimizing contamination with gloves and sterile razor blades, catheters were cut into 1-inch pieces, placed in polypropylene tubes, and stored at −20°C. Urine samples were obtained from catheter bag ports swabbed clean with alcohol prior to collection. Urine aliquots of 20–50 mL were stored at −20°C. We could not rule out infrequent draining of catheter bags and *ex vivo* microbial growth in UP specimens. Matching CB and UP samples were not expected to have identical microbial contents. All specimens were kept frozen during transport and transferred to a −80°C freezer until further use.

### Urine and Catheter Specimen Extraction and Protein Solubilization for Proteomics

The catheter materials were latex in the case of eight patients and silicone (patient P7). The color and turbidity of urine specimens and salt crystallization inside and on the surface of catheter pieces were noted. To obtain a UP from a sample, an aliquot was thawed, adjusted to 20°C, and, if acidic, neutralized with 1 M Tris–HCl (pH 8.1) to a pH of ~6.5 to 7.5 and centrifuged at 3,200 *g* for 15 min. UPs were aliquoted for rDNA and protein extractions and spare samples in ratios of ~10, 45, and 45%, respectively. Urethral catheter pieces were extracted in two steps. Submerged in 100 mM sodium acetate (pH 5.5), 20 mM sodium metaperiodate, and 300 mM NaCl, catheter pieces were agitated in an ultrasonic water bath for 10 min at 20°C. The supernatants were recovered and concentrated followed by two solubilization cycles of the pellets with a denaturing SED solution [1% sodium dodecyl sulfate (SDS) (vol/vol), 5 mM EDTA and 50 mM DTT] including 3-min heat treatment at 95°C. Two supernatants were recovered that we termed CB_1_ (Na-acetate buffer) and CB_2_ (SED solution). CB_1_ and CB_2_ fractions were separately analyzed *via* LC-MS/MS. Raw data were merged for database searches as described in the following section. UP samples were solubilized in SED solution as described previously (13, 27). Centrifugation steps were performed using Ultrafree-4 filter units (10 kDa MWCO) to collect large peptides and proteins in the concentrates. Peptides with low *M*_r_ values such as neutrophil defensin 1 (4 kDa) were also retained. Solubilized UP and CB samples were subjected to filter-aided sample preparation (FASP) (55) adapted to 100 μg protein for digestion with sequencing-grade trypsin (50:1 ratio) in Vivacon 10k filters (Sartorius AG, Germany) (13). FASP-processed peptide mixtures were desalted using the Stage-Tip method (56) and lyophilized for LC-MS/MS analysis.

### Shotgun Proteomics *via* LC-MS/MS

Desalted peptide mixtures derived from UP, CB_1_, and CB_2_ samples were dissolved in 10 μL 0.1% formic acid (solvent A) and analyzed using one of two LC-MS/MS systems: (1) a high-resolution Q-Exactive mass spectrometer (MS) coupled to an Ultimate 3000-nano LC system; (2) an LTQ-Velos Pro ion-trap MS coupled to the Easy-nLC II system (each Thermo Scientific, San Jose, CA). Each system was equipped with a FLEX nano-electrospray ion source at the LC-MS interface. Detailed LC-MS/MS procedures were described for the Q-Exactive (57, 58) and LTQ-Velos Pro (59) platforms. Briefly, electrospray ionization was achieved by applying 2.0 kV distally *via* a liquid junction. For Velos Pro analysis, longer LC gradients were applied. The peptides present in samples were trapped on a C_18_ trap column (100 μm × 2 cm, 5-μm pore size, 120 Å) and separated on a PicoFrit C_18_ analytical column (75 μm ×15 cm, 3-μm pore size, 150 Å) at a flow rate of 200 nL/min. Starting with solvent A, a linear gradient from 10 to 30% solvent B (0.1% formic acid in acetonitrile) over 195 min was followed by a linear gradient from 30 to 80% solvent B over 20 min and column re-equilibration with solvent A for 5 min. Columns were washed thrice using a 30-min solvent A to B linear gradient elution to avoid sample carryover. Peptide ions were analyzed in an MS^1^ data-dependent mode to select ions for MS^2^ scans using the software application XCalibur *v2.2* (Thermo Scientific). The fragmentation modes were collision-induced dissociation with a normalized collision energy of 35% (LTQ-Velos Pro) and high-energy collisional dissociation with a normalized collision energy of 27% (Q-Exactive). Dynamic exclusion was enabled. MS^2^ ion scans for the same MS^1^ *m/z* value were repeated once and then excluded from further analysis for 30 s. Survey (MS^1^) scans ranged from *m/z* range of 380 to 1,800 followed by MS^2^ scans for selected precursor ions. Survey scans with the Q-Exactive were acquired at a resolution of 70,000 (m/Δm) with *m/z* range from 250 to 1,800. MS^2^ scans were performed at a resolution of 17,500. The 10 most intense ions were fragmented in each cycle. Unassigned ions and those with a charge of +1 were rejected from further analysis. Two or three LC-MS/MS replicates were run for UP, CB_1_, and CB_2_ sample extracts. Samples for all eight patients were analyzed using the LTQ-Velos Pro system. P2 and P8 samples were also processed using the Q-Exactive LC-MS/MS platform. Dual proteomic analyses—from each of the LC-MS/MS systems—allowed data comparisons. Using the Proteome Discoverer v1.4 software tool (Thermo Scientific), the identification and quantification data were similar for highly to moderately abundant proteins in the matching datasets. Given that we had LTQ-Velos Pro data for all samples, we used those results for differential quantitative analyses.

### Computational Proteomic Data Analyses

The raw MS files were combined for database searches as follows: (1) the two to three replicates for a UP sample; (2) the two to three replicates from both CB_1_ and CB_2_ fractions for a CB sample. The Sequest HT algorithm integrated in Proteome Discoverer v1.4 was used as the search engine with analytical parameters described previously (57, 58). Only rank-1 peptides with a length of at least seven amino acids were considered. The false discovery rate (FDR) rates were estimated using the integrated Percolator tool with a (reverse sequence) decoy database. Protein hits identified with a 1% FDR threshold were accepted for data interpretation. The “protein grouping” function was enabled to ensure that one protein was reported when multiple proteins shared a set of identified peptides. Database contents were the reviewed protein sequence entries in the non-redundant human UniProt database (release 2015-16; 20,195 sequences), complete sequence entries for 23 genomes of microbes reported to cause UTI (57), and 28 complete sequence entries for bacterial genomes rarely associated with UTI, guided by genus identification data from 16S rRNA analyses. Separate searches using sequence databases for species part of the same genus were conducted to select those species that had the highest number of unique proteins identifications according to PSMs for each data series from a given patient. This was particularly important for members of the Enterobacteriaceae family given that their protein sequence similarities are high and that they were the most frequent CB residents. The microbial species for the database searches are listed in Supplementary Table 1. Pan-proteome database searches for *E. coli* and *P. mirabilis* were performed by creating non-redundant sequence databases using CD-Hit with a 95% sequence identity threshold, eliminating matched open reading frames (ORFs) from several annotated *E. coli* and *Proteus* genomes. All MS raw files were deposited in PRIDE (*via* ProteomeXchange) under the data identifier PXD012048. PSM counts assigned to each microbial species separately, and *Homo sapiens* were the basis for species-based quantification to assess their contributions to the total proteome of a given sample. To measure the contribution of each protein assigned to a given bacterial species to the proteome in a sample, we divided the protein's PSMs by the sum of all PSMs for that species in that sample. This normalization step enabled comparisons of individual proteins, or that part of complexes and functional clusters, regardless of how abundant that species was in the respective polymicrobial proteome. These data were also derived from the Proteome Discoverer v1.4 datasets. This normalization was also applied to data for human proteomes. Postnormalization quantities are termed *proteomic abundance units* and are applied in the *y* axes of the plots in Figures 5–8 and in Supplementary Material.

### Microbial Taxonomy Analyses

Microbial cell lysis and DNA extraction methods from catheter extracts and UP samples (27) and the amplification of V1–V3 regions of the 16S rDNA bacterial genes as well as sequencing on the MiSeq sequencer from Illumina Inc. were described previously (60). The UPARSE pipeline for the phylogenetic analysis was used (61). Operational taxonomic units (OTUs) were generated *de novo* from raw sequence reads using default parameters in UPARSE, the Wang classifier and bootstrapping using 100 iterations. The taxonomies were assigned to the OTUs using Mothur and applying the SILVA 16S rRNA database version 123 as reference database (62). Unbiased, metadata-independent filtering was applied at each level of the taxonomy by eliminating samples with fewer than 2,000 reads and OTUs present in fewer than 10 samples. Filtered data were analyzed based on the relative contributions of microbial genera in a distinct sample. The Shannon index was used to measure the alpha diversity.

### Microbial Cultures

Microbial culture data are only tangentially described here. We refer to previous publications for methods used to culture bacteria anaerobically and recover fastidious bacterial organisms (27, 34). Other bacterial and *C. albicans* isolates were regrown from phosphate-buffered saline (PBS) extracts of catheters following storage at −80°C. Such cultures were performed for 20–72 h on rich agar media containing BHI broth, MacConkey media, or blood at 30°C using ambient conditions or incubators with 5% CO_2_. Microbial colonies were examined both microscopically and *via* LC-MS/MS to assess for purity of a colony isolate or a liquid culture. Bacterial stocks were stored in rich growth media containing 10% glycerol quickly freezing at −80°C.

### Western Blot Experiments

Proteins in 10–20 μg total amounts from UP sample lysates were applied for Western blot experiments, first separating proteins in an SDS–polyacrylamide gel electrophoresis gel (4–12% T acrylamide concentration) and then electroblotting proteins onto a polyvinylidene fluoride membrane and finally applying antibody to incubation and chemiluminescent detection experiments. The methods were previously described (10). Anti-MPO heavy-chain C-16 polyclonal antibodies raised against MPO peptide segments (product # sc-16128-R; Sta. Cruz Biotechnology Inc.) diluted 1:1,000 in PBS were antibodies for the first incubation step. A horseradish peroxidase conjugate of goat anti-rabbit immunoglobulin G–horseradish peroxidase (sc-2004) diluted 1:5,000 was used for the second incubation step. Exposure to chemiluminescent substrates was completed once the bands emerged (1–5 min) using an ImageQuant LAS 500 chemiluminescence CCD camera.

### Statistical Analysis Methods

Wilcoxon rank sum tests were performed to elucidate statistical changes in taxonomic α-diversity. Analysis of variance was applied to compare the variation of biomasses for CB data comparing patients. We applied a *P*-value cutoff of 0.05 to determine statistical significance. Hierarchical clustering was performed using the proteomic analysis software Perseus v1.6.0.2. We used the Pearson correlation (with complete linkage mode) to identify gene and patient clusters *via* PCA or in heat maps. The non-parametric Kruskal–Wallis *H* and Mann–Whitney *U*-tests were used to determine the statistical differences in abundance of proteins comparing abundance data series from more than 2 and 2 patients, respectively. For systems composed of multiple protein subunits or contributing to functional contexts (e.g., oxidative stress response and MAF), PSMs of the proteins were summed prior to normalization. Box plots were generated in GraphPad Prism software.

## Data Availability Statement

The datasets presented in this study can be found in online repository ProteomeXchange via identifier PXD012412.

## Ethics Statement

The studies involving human participants were reviewed and approved by Western Institutional Review Board (WIRB) and J. Craig Venter Institutional Review Board. The patients/participants provided their written informed consent to participate in this study.

## Author Contributions

YY major contributions to experimental data generation, proteomic data analysis, and manuscript writing. HS major contributions to data analysis and manuscript writing. TT sample preparation and organization. PS sample preparation and extensive manuscript review. SB sample preparation. Y-HL protein experiment analysis. KM metagenomics sample preparation. MT metagenomics experiment design. LM clinical study design and medical data collection. RW clinical study design. KN study design and manuscript review. RP study conceptualization, study design, data analysis, and manuscript writing. All authors contributed to the article and approved the submitted version.

## Conflict of Interest

The authors declare that the research was conducted in the absence of any commercial or financial relationships that could be construed as a potential conflict of interest.
