# Supplementary material for: Urethral Catheter Biofilms Reveal Plasticity in Bacterial Composition and Metabolism and Withstand Host Immune Defenses in Hypoxic Environment
Source: Front Med (Lausanne). 2021 Jun 23;8:667462. doi: 10.3389/fmed.2021.667462 (PMC8260951; doi:10.3389/fmed.2021.667462)
Supplement: Supplementary file 1 [file Data_Sheet_1.ZIP › Supplementary Materials folder/Supplementary Table S1.docx]

| **Supplementary Table 1**. Metaproteomic database searches utilized entire genome sequence-derived protein sequence entries (ORFs) from subsets of 49 microbial species (strains) and Homo sapiens (databases as of July 2019). | | | | |
| --- | --- | --- | --- | --- |
| **#** | **Microbial species and strain** | **UniProt web link** | **Number of ORFs / annotated proteins** | **Taxon identifier** |
| 1 | Acidovorax citrulli (strain AAC00-1) (Acidovorax avenae subsp. citrulli) | http://www.uniprot.org/taxonomy/397945 | 4,603 | 397945 |
| 2 | Acinetobacter baumannii BIDMC 57 | http://www.uniprot.org/taxonomy/1439317 | 3,764 | 1439317 |
| 3 | Actinobaculum massiliense ACS-171-V-Col2 | http://www.uniprot.org/taxonomy/883066 | 1,696 | 883066 |
| 4 | Actinotignum schaalii FB123-CNA-2 | http://www.uniprot.org/taxonomy/883067 | 1,726 | 883067 |
| 5 | Aerococcus urinae (strain ACS-120-V-Col10a) | http://www.uniprot.org/taxonomy/866775 | 1,684 | 866775 |
| 6 | Anaerococcus vaginalis ATCC 51170 | http://www.uniprot.org/taxonomy/655811 | 1,764 | 655811 |
| 7 | Bacteroides fragilis (strain 638R) | http://www.uniprot.org/taxonomy/862962 | 4,284 | 862962 |
| 8 | Bifidobacterium scardovii | http://www.uniprot.org/taxonomy/158787 | 2,496 | 158787 |
| 9 | Bordetella hinzii | http://www.uniprot.org/taxonomy/103855 | 4,574 | 103855 |
| 10 | Bordetella pertussis (strain Tohama I / ATCC BAA-589 / NCTC 13251) | http://www.uniprot.org/taxonomy/257313 | 3,260 | 257313 |
| 11 | Brevundimonas diminuta (strain ATCC 11568 / DSM 7234 / NBRC 12697 / NCIMB 9393 / NCTC 8545) | http://www.uniprot.org/taxonomy/751586 | 3,002 | 751586 |
| 12 | Campylobacter jejuni subsp. jejuni serotype O:2 (strain ATCC 700819 / NCTC 11168) | http://www.uniprot.org/taxonomy/192222 | 1,624 | 192222 |
| 13 | Candida albicans (strain WO-1) | http://www.uniprot.org/taxonomy/294748 | 5,742 | 294748 |
| 14 | Citrobacter koseri (strain ATCC BAA-895 / CDC 4225-83 / SGSC4696) | http://www.uniprot.org/taxonomy/290338 | 5,020 | 290338 |
| 15 | Citrobacter rodentium (strain ICC168) | http://www.uniprot.org/taxonomy/637910 | 4,792 | 637910 |
| 16 | Cloacibacterium normanense | http://www.uniprot.org/taxonomy/237258 | 2,558 | 237258 |
| 17 | Clostridium thermocellum (strain ATCC 27405 / DSM 1237 / NBRC 103400 / NCIMB 10682 / NRRL B-4536 / VPI 7372) | http://www.uniprot.org/taxonomy/203119 | 3,107 | 203119 |
| 18 | Corynebacterium urealyticum (strain ATCC 43042 / DSM 7109) | http://www.uniprot.org/taxonomy/504474 | 2,011 | 504474 |
| 19 | Enterobacter cloacae subsp. cloacae (strain ATCC 13047 / DSM 30054 / NBRC 13535) | http://www.uniprot.org/taxonomy/716541 | 5,411 | 716541 |
| 20 | Enterococcus faecalis (strain ATCC 700802 / V583) | http://www.uniprot.org/taxonomy/226185 | 3,240 | 226185 |
| 21 | Enterococcus faecium 10/96A | http://www.uniprot.org/taxonomy/1391465 | 3,261 | 1391465 |
| 22 | Escherichia coli (strain UTI89 / UPEC) | http://www.uniprot.org/taxonomy/364106 | 5,192 | 364106 |
| 23 | Finegoldia magna (strain ATCC 29328) | http://www.uniprot.org/taxonomy/334413 | 1,814 | 33413 |
| 24 | Fusobacterium nucleatum subsp. nucleatum (strain ATCC 25586 / CIP 101130 / JCM 8532 / LMG 13131) | http://www.uniprot.org/taxonomy/190304 | 2,050 | 190304 |
| 25 | Gardnerella vaginalis (strain ATCC 14019 / 317) | http://www.uniprot.org/taxonomy/525284 | 1,365 | 525284 |
| 26 | Globicatella sp. HMSC072A10 | http://www.uniprot.org/taxonomy/1739315 | 2,027 | 1739315 |
| 27 | Haemophilus influenzae (strain ATCC 51907 / DSM 11121 / KW20 / Rd) | http://www.uniprot.org/taxonomy/71421 | 1,710 | 71421 |
| 28 | Klebsiella aerogenes (strain ATCC 13048 / DSM 30053 / JCM 1235 / KCTC 2190 / NBRC 13534 / NCIMB 10102 / NCTC 10006) (Enterobacter aerogenes) | http://www.uniprot.org/taxonomy/1028307 | 4,910 | 1028307 |
| 29 | Klebsiella oxytoca (strain ATCC 8724 / DSM 4798 / JCM 20051 / NBRC 3318 / NRRL B-199 / KCTC 1686) | http://www.uniprot.org/taxonomy/1006551 | 5,484 | 1006551 |
| 30 | Klebsiella pneumoniae (strain 342) | http://www.uniprot.org/taxonomy/507522 | 5,738 | 507522 |
| 31 | Lactobacillus gasseri (strain ATCC 33323 / DSM 20243) | http://www.uniprot.org/taxonomy/324831 | 1,694 | 324831 |
| 32 | Listeria monocytogenes serovar 1/2a (strain ATCC BAA-679 / EGD-e) (Strain: ATCC BAA-679 / EGD-e) | http://www.uniprot.org/taxonomy/169963 | 2,860 | 169963 |
| 33 | Morganella morganii subsp. morganii KT | http://www.uniprot.org/taxonomy/1124991 | 3,510 | 1124991 |
| 34 | Mycoplasma genitalium (strain ATCC 33530 / G-37 / NCTC 10195) | http://www.uniprot.org/taxonomy/243273 | 484 | 243273 |
| 35 | Pasteurella multocida (strain Pm70) | http://www.uniprot.org/taxonomy/272843 | 2,016 | 272843 |
| 36 | Prevotella melaninogenica D18 | http://www.uniprot.org/taxonomy/575612 | 2,461 | 575612 |
| 37 | Propionimicrobium lymphophilum ACS-093-V-SCH5 | http://www.uniprot.org/taxonomy/883161 | 2,076 | 883161 |
| 38 | Proteus mirabilis (strain HI4320) | http://www.uniprot.org/taxonomy/529507 | 3,661 | 529507 |
| 39 | Providencia stuartii (strain MRSN 2154) | http://www.uniprot.org/taxonomy/1157951 | 4,219 | 1157951 |
| 40 | Pseudomonas aeruginosa (strain PA7) | http://www.uniprot.org/taxonomy/381754 | 6,246 | 381754 |
| 41 | Rhodanobacter denitrificans | http://www.uniprot.org/taxonomy/666685 | 3,777 | 666685 |
| 42 | Serratia marcescens WW4 | http://www.uniprot.org/taxonomy/435998 | 4,801 | 435998 |
| 43 | Staphylococcus aureus (strain USA300 / TCH1516) | http://www.uniprot.org/taxonomy/451516 | 2,694 | 451516 |
| 44 | Staphylococcus epidermidis (strain ATCC 35984 / RP62A) | http://www.uniprot.org/taxonomy/176279 | 2,493 | 176279 |
| 45 | Staphylococcus saprophyticus subsp. saprophyticus (strain ATCC 15305 / DSM 20229) | http://www.uniprot.org/taxonomy/342451 | 2,404 | 342451 |
| 46 | Stenotrophomonas maltophilia (strain K279a) | http://www.uniprot.org/taxonomy/522373 | 4,367 | 522373 |
| 47 | Streptococcus agalactiae serotype V (strain ATCC BAA-611 / 2603 V/R) | http://www.uniprot.org/taxonomy/208435 | 2,105 | 208435 |
| 48 | Trueperella bernardiae | http://www.uniprot.org/taxonomy/59561 | 1,770 | 59561 |
| 49 | Veillonella parvula (strain ATCC 10790 / DSM 2008 / JCM 12972 / Te3) | http://www.uniprot.org/taxonomy/479436 | 1,846 | 479436 |
| 50 | Homo sapiens (Reviewed) | http://www.uniprot.org/taxonomy/9606 | 20,259 | 9606 |
