# Supplementary material for: Urethral Catheter Biofilms Reveal Plasticity in Bacterial Composition and Metabolism and Withstand Host Immune Defenses in Hypoxic Environment
Source: Front Med (Lausanne). 2021 Jun 23;8:667462. doi: 10.3389/fmed.2021.667462 (PMC8260951; doi:10.3389/fmed.2021.667462)
Supplement: Supplementary file 1 [file Data_Sheet_1.ZIP › Supplementary Materials folder/Legends Supplementary Figuers 1-5.docx]

**Legends for Supplementary Figures 1-5.**

Supplementary Figure 1 (images denoted Sup Fig 1a and 1b). Heat maps of human proteomes derived from urinary pellet (UP) samples (1a) and catheter biofilm (CB) samples (1b). Hierarchical clustering analyses using Pearson Correlation (complete linkage) were performed for samples (top panel) and proteins (to the left of the heat map). In 1a, proteomes of UP samples associated with short-term catheterization were included in the analysis (terms “d1” and “d3” are used to indicate duration of catheterization in days). The profiles had evidence of bacteriuria. In 1b, a short red bar on the left between dendrogram and heat map indicates a subcluster of proteins rich in neutrophil and eosinophil granule proteins.

Supplementary Figure 2 (image denoted Sup Fig 2). Correlation analysis for neutrophil and eosinophil granule proteins from UP datasets. Correlation analysis for neutrophil and eosinophil granule proteins from UP datasets using the human proteome data in Suppl. Datasheet 1 from all patients. The correlation coefficient is shown in each box. PERE (EPX) and PRG2 are eosinophil granule proteins. TRFL (LTF), PERM (MPO), CATG (CTSG), ENLE (NE), CAP7 (AZU) are enriched in azurophilic granules. Other groups of proteins with high correlation coefficients are MMP9, LKHA4, PADI4 (neutrophil granule effectors) and PGRP1, BPI, MMP8 and RETN (also neutrophil granule effectors). The protein acronyms reflect UniProt short names.

Supplementary Figure 3 (image denoted Sup Fig 3). Microbial proteome analyses of extracts from longitudinally collected catheter biofilm specimens of four patients. The sampling extended over 12 weeks (Patient 4, P4), 25 weeks (Patient 5, P5), 23 weeks (Patient 8, P8), and 24 weeks (Patient 9, P9). The microbial organisms are mostly listed at the species level, but only at the genus level when genome information was restricted to few species. The bars represent serially replaced catheters (“CB”) from left to right, with occasionally matching urinary pellet “UP” samples displayed separately. Differently colored bar segments represent the estimated microbial quantity based on peptide-spectral counts for a given species in a given proteomic dataset. The entire bar height represents the fraction of microbial PSM quantity over total PSM quantity (human proteins were identified in each sample). As indicated, patient P9 was systemically treated with levofloxacin at the indicated timepoint due to a wound infection (timepoint is depicted twice because UP and CB samples are separated in the bar diagrams).

Supplementary Figure 4 (image denoted Sup Fig 4). Analysis of bacterial abundances in a comparison of UP and CB samples from matched time points pathogens in box plots. The analyses were conducted separately for species recurring in proteomic profiles from a variable number of patients. In none of the cases were the abundance differences statistically significant suggesting that bacteria randomly disperse from catheter biofilms.

Supplementary Figure 5 (images denoted Sup Fig 5a and 5b). Hierarchical clustering analyses for *E. coli* and *P. mirabilis* proteome datasets derived from four and five patients, respectively. In these data series there was good to excellent coverage of the respective proteomes in at least 4 different CBs per patient. In 5a, data for *E. coli* proteomes (600 selected proteins). Patients are identified as P1, P2, P 5, and P8. Proteome datasets derived from LC-MS/MS analysis of urinary pellet (UP) and catheter biofilm (CB) extracts are listed with the numerical identifier, adding the type of samples: UP and CB. The higher the number for a given patient, the later the specimen collection timepoint. The exception was UP16 which was collected after UP2 and before UP3. In 5b, data for *P. mirabilis* proteomes (700 selected proteins). Patients are identified as P1, P4, P 5, P6, and P7. Proteome datasets derived from LC-MS/MS analysis of the extracts have terms defined above for *E. coli*. We observed that proteomic data for a given patient often clustered.
