# Supplementary figures and images for: Urethral Catheter Biofilms Reveal Plasticity in Bacterial Composition and Metabolism and Withstand Host Immune Defenses in Hypoxic Environment
Source: Front Med (Lausanne). 2021 Jun 23;8:667462. doi: 10.3389/fmed.2021.667462 (PMC8260951; doi:10.3389/fmed.2021.667462)

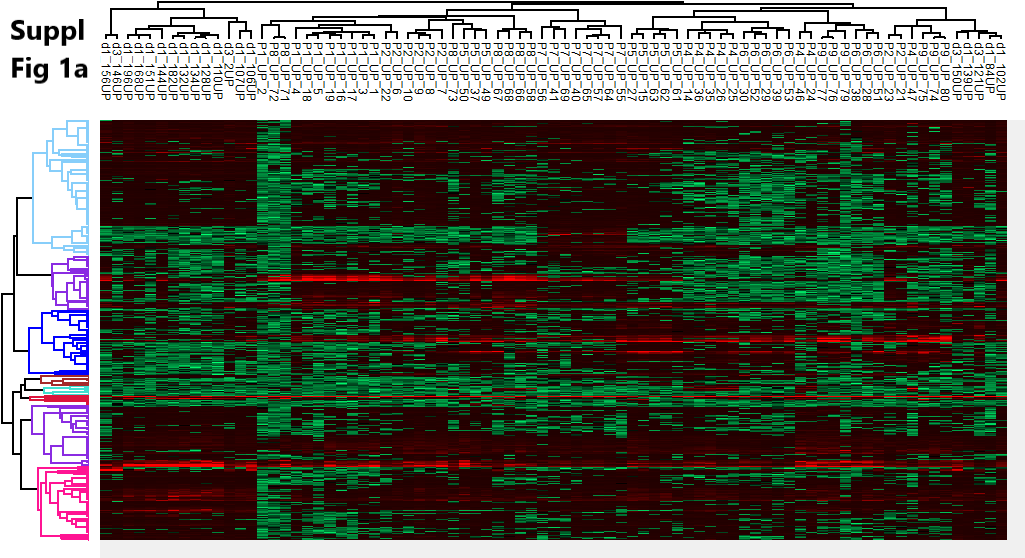

Supplement: Supplementary file 1 [file Data_Sheet_1.ZIP › Supplementary Materials folder/Supplementary Figure 1a.png]

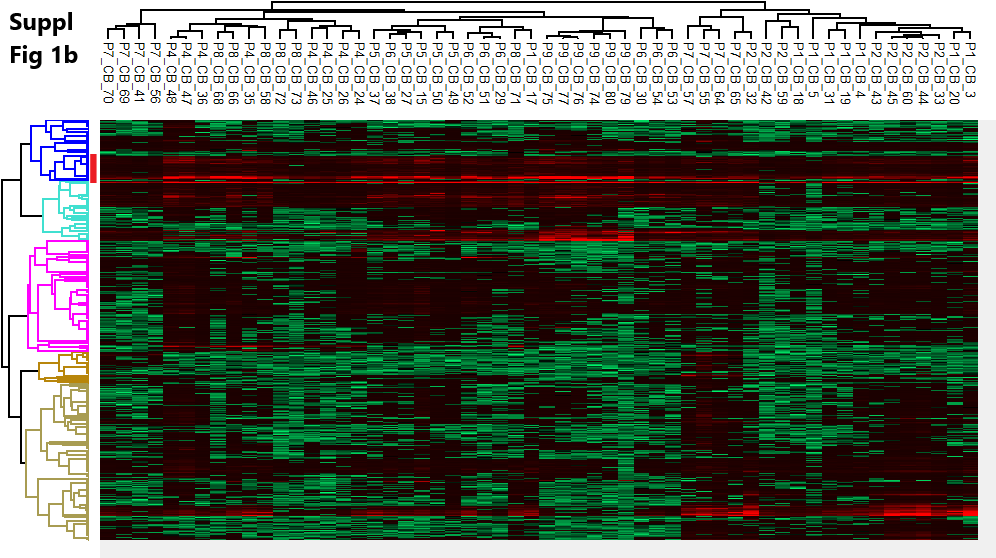

Supplement: Supplementary file 1 [file Data_Sheet_1.ZIP › Supplementary Materials folder/Supplementary Figure 1b.tiff]

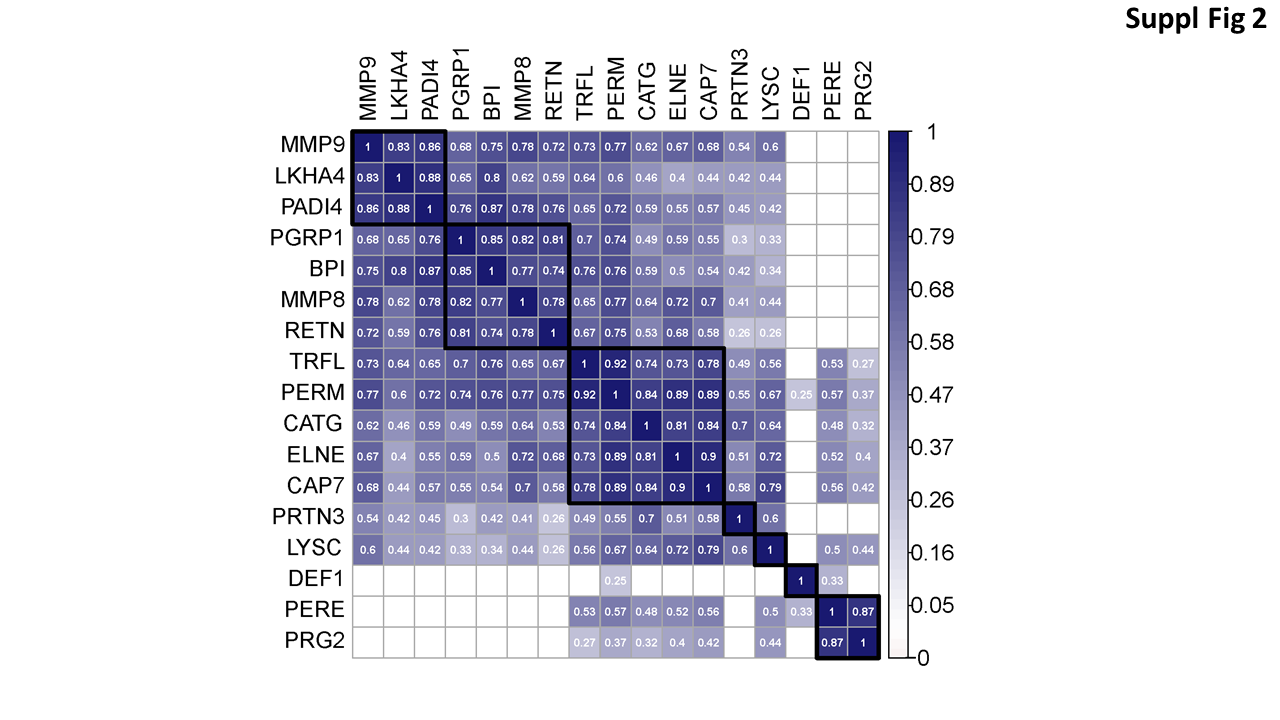

Supplement: Supplementary file 1 [file Data_Sheet_1.ZIP › Supplementary Materials folder/Supplementary Figure 2.tif]

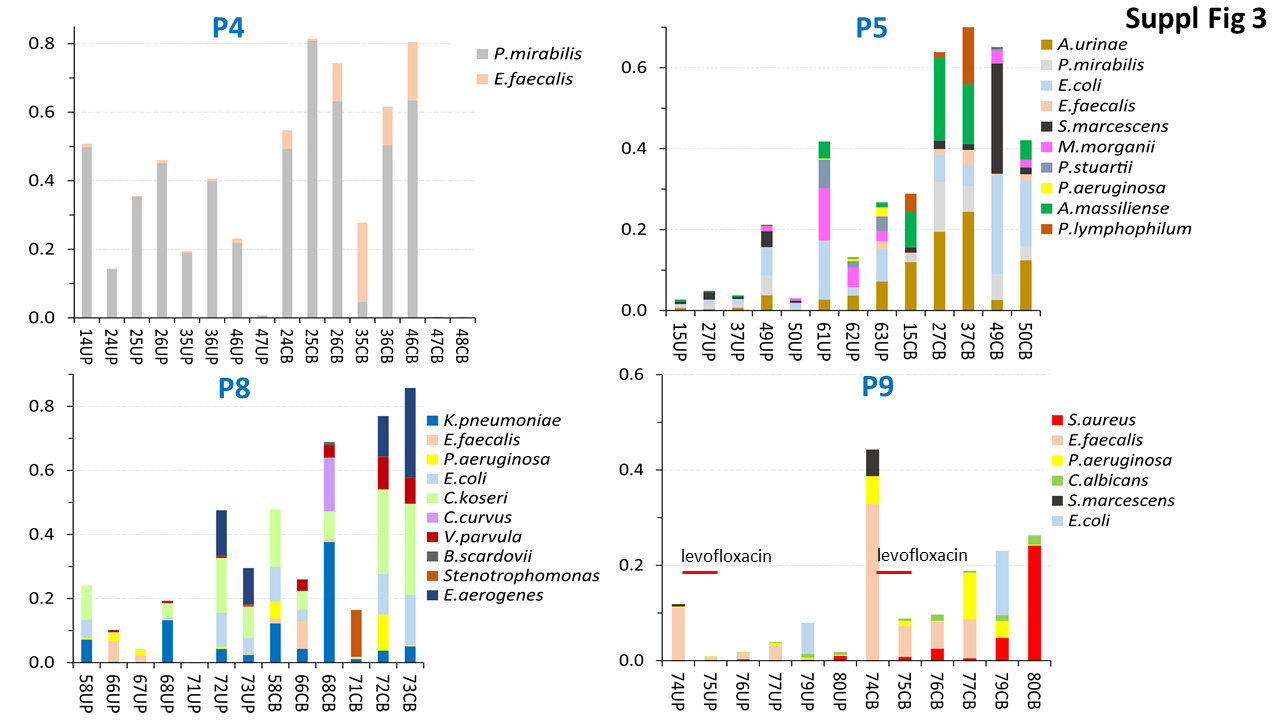

Supplement: Supplementary file 1 [file Data_Sheet_1.ZIP › Supplementary Materials folder/Supplementary Figure 3.tif]

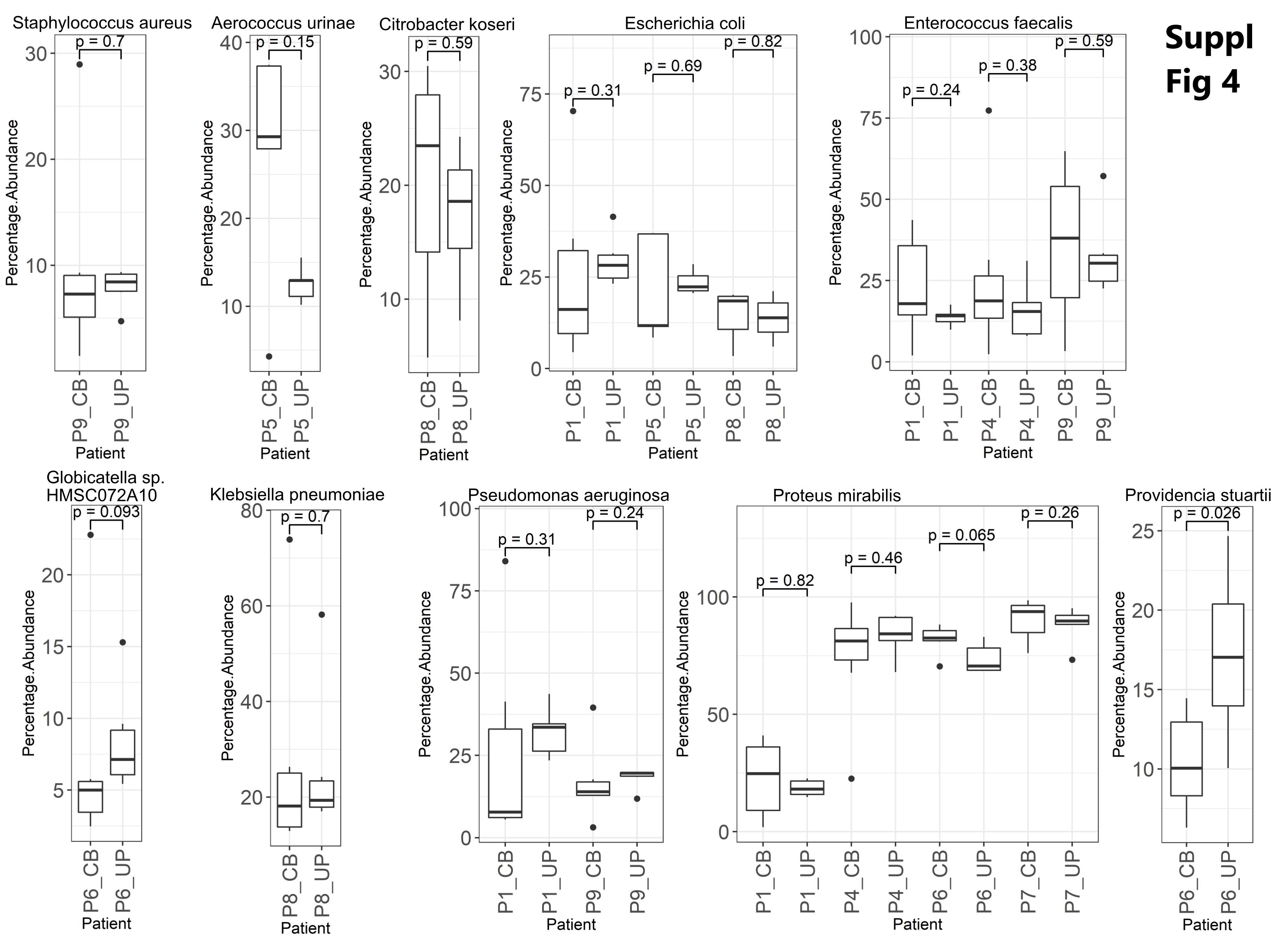

Supplement: Supplementary file 1 [file Data_Sheet_1.ZIP › Supplementary Materials folder/Supplementary Figure 4.png]

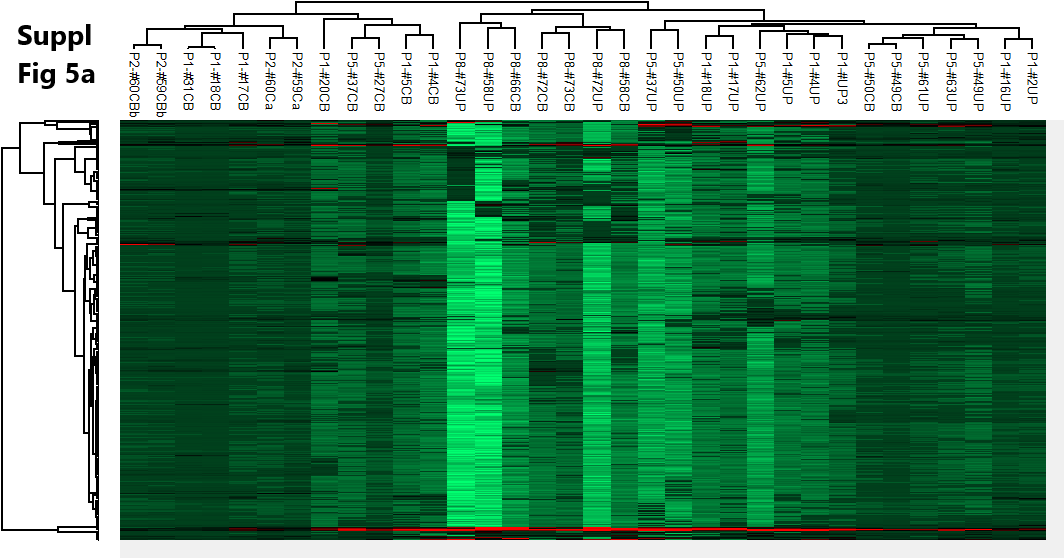

Supplement: Supplementary file 1 [file Data_Sheet_1.ZIP › Supplementary Materials folder/Supplementary Figure 5a.png]

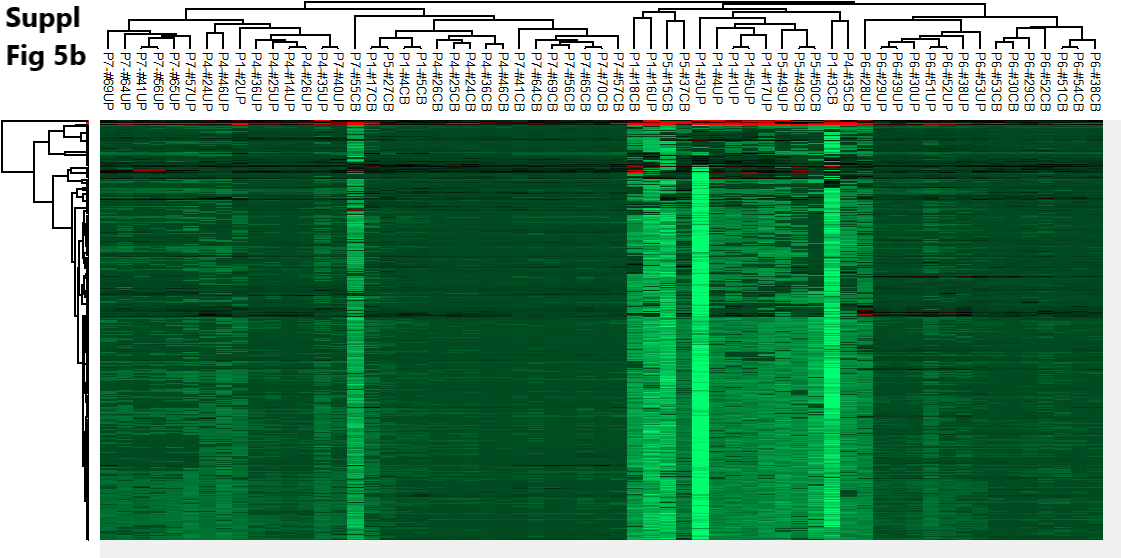

Supplement: Supplementary file 1 [file Data_Sheet_1.ZIP › Supplementary Materials folder/Supplementary Figure 5b.png]
